# Adaptation of the Quick Aphasia Battery into Urdu and establishment of its psychometric properties

**DOI:** 10.1371/journal.pone.0356908

**Published:** 2026-09-01

**Authors:** Abeeha Fatima, Anum Ashraf, Muhammad Azzam Khan

**Affiliations:** 1 Department of Pediatric Physical Therapy and Neurorehabilitation, University of Lahore Teaching Hospital, Lahore, Punjab, Pakistan; 2 Department of Rehabilitation Sciences, The University of Lahore, Lahore, Punjab, Pakistan; 3 Department of Allied Health Science and Practice, University of Adelaide, Adelaide, South Australia, Australia; University of Thessaly Faculty of Medicine: Panepistemio Thessalias Tmema Iatrikes, GREECE

## Abstract

Quick Aphasia Battery provides a quick yet comprehensive evaluation of language performance in individuals with aphasia. While the tool was originally developed in English, no validated Urdu version existed for use in Pakistani clinical settings. This study aimed to translate and culturally adapt the Urdu version of the Quick Aphasia Battery and determine its psychometric properties, including reliability and preliminary diagnostic performance among stroke patients. The study was conducted in three phases. Phase 1 involved translation and adaptation, comprising forward-backward translation, expert review, cultural modification and pilot testing with 20 participants to assess clarity and feasibility. Phase 2 was validation including 44 participants (7 with aphasia and 37 without aphasia) to establish content validity, construct validity, and preliminary diagnostic performance. Phase 3 evaluated reliability, including internal consistency, inter-rater, test-retest and parallel forms reliability. Content validity indices were excellent (I-CVI = 0.75–1.00; S-CVI = 0.95–1.00). Mann-Whitney U test revealed significant group differences across all subtests (p < 0.001), supporting known group validity. The Preliminary Receiver Operating Characteristic analysis demonstrated an AUC of 0.96 (95% CI: 0.90–0.99), with a sensitivity of 86% and specificity of 92%. Reliability testing indicated inter-rater reliability with an Intraclass Correlation Coefficient value of 0.96, test-retest reliability with a value of 0.98 and internal consistency with a Cronbach’s α value of 0.96; there was no significant difference across parallel forms (p = 0.526). The Urdu Quick Aphasia Battery exhibits promising preliminary validity and reliability for assessing language performance in Urdu-speaking stroke patients and shows consistency and agreement with the original version, despite the inclusion of culturally adapted modifications. However, given the limited number of aphasia cases, further validation with larger clinical samples is required before establishing its diagnostic utility.

## Introduction

South Asia is home to densely populated countries, with Pakistan being the fifth most populous in the world [1]. Urbanization has spurred socioeconomic changes, resulting in better control of infectious diseases, lower mortality rates, and increased life expectancy. Consequently, there is an increased risk of age-related illnesses, including cardiac disease and stroke. Higher rates of diabetes and hypertension, even among younger individuals, have led to a rise in small artery occlusion ischemic strokes among South Asians [2]. Stroke is a common cause of aphasia, with approximately 1/3 of stroke survivors experiencing some form of language impairment [3].

Stroke incidence has decreased by 42% in high-income countries; it has alarmingly doubled in low-to-middle-income countries [4]. There is a scarcity of population-based data on stroke epidemiology in South Asian countries, including Pakistan [5]. Consequently, public awareness and understanding of stroke risk factors and management are very limited. Approximately 21–38% of patients who suffer a stroke are diagnosed with aphasia, a common complication resulting in language impairment [6]. This condition can affect various aspects of language, including phonological, morphological, semantic, syntactic, pragmatic, and motoric functions of speech [7]. There are different types of aphasia, and the specific symptoms can vary depending on the location and extent of the brain damage [8].

Speech-Language Pathologists employ a variety of assessment methods to diagnose aphasia comprehensively. Initially, they gather a detailed case history and conduct patient interviews to understand the individual’s medical background, symptom onset, and overall health. Standardized aphasia tests, such as the Boston Diagnostic Aphasia Examination (BDAE), Western Aphasia Battery (WAB), and Comprehensive Aphasia Test (CAT), are frequently used to evaluate language skills across multiple domains, including conversational speech, auditory comprehension, oral expression, reading, and writing [9].

Assessing individuals with aphasia in their native language is important for ensuring diagnostic accuracy and culturally appropriate interpretation. When assessment is carried out in any other language, the results may be affected by factors such as limited language proficiency, unfamiliar vocabulary, or psycholinguistic differences rather than the true effects of aphasia, which can lead to misdiagnosis and inappropriate treatment. Native language assessment allows clinicians to diagnose a patient’s actual linguistic abilities, supports natural communication behaviors, and enhances the patient’s comfort and participation during assessment. In addition, culturally and linguistically adapted tools are crucial for equitable access to care and personalized rehabilitation outcomes [10].

Internationally, a variety of aphasia tests exist. These tools are well validated; however, they often require 60–90 minutes to administer, limiting their practicality in acute care and in resource-constrained settings [11]. In Pakistan, some progress has been made with the development of localized assessments such as Bilingual Aphasia Test (BAT) in Urdu and Urdu Aphasia Assessment Tool (UAAT) and a specific aphasia screener in Urdu. These represent important steps forward, yet challenges remain regarding administration time, cross-cultural validation, and consistent clinical adaptation [12].

The Quick Aphasia Battery (QAB) is a short and reliable test designed to evaluate language performance in people with aphasia, most often after a stroke or other neurological injury. Unlike longer tools such as the BDAE or the WAB, which can take 1–2 hours, the QAB can be administered in 15–25 minutes and still gives a detailed profile of the person’s strengths and weaknesses. It consists of eight subtests that measure different aspects of language such as comprehension, naming, repetition, connected speech, reading, and motor speech using structured tasks and stimulus cards. Each subtest is scored on a simple 0–4 scale, and the combined scores give an overall QAB score that reflects the severity of aphasia. Because of its shorter time administration and detailed subtests, the QAB has become widely used in both clinical practice and research, particularly in acute stroke settings [13].

Cross-linguistic validation in Chinese, Turkish, Arabic, Lebanese, French, and Spanish has confirmed its reliability and diagnostic accuracy [14].

This study aimed to adapt the QAB into Urdu and determine its psychometric properties. The Urdu adaptation of the QAB is therefore timely and essential, offering clinicians a quick, standardised, and psychometrically robust tool. By addressing the gap between lengthy internationally developed batteries and limited local tools, the QAB-Urdu adaptation has the potential to improve clinical care and support evidence-based practice for people with aphasia in Pakistan.

## Materials and Methods

**Fig 1:**
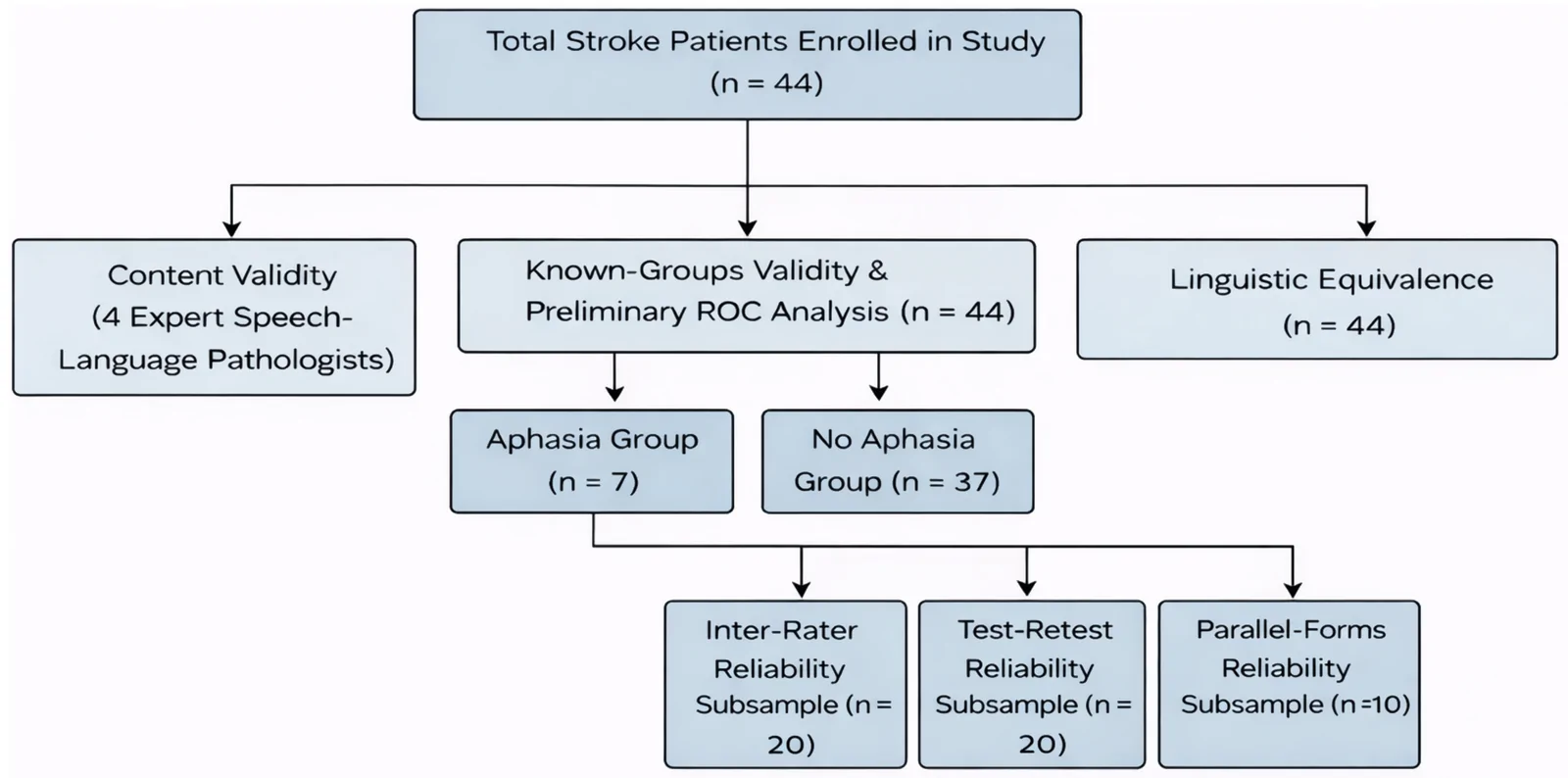
Flow of participants across different phases.

The present study utilised a quantitative, cross-sectional design to adapt and validate the QAB into Urdu. The study was carried out in three major phases: 1) translation and adaptation, 2) validity, and 3) reliability testing. Before the adaptation process began, formal permission was obtained from the original author of the QAB. The permission record has been attached as supporting information (S2 File Author permission). Ethical approval was obtained from the Institutional Review Board of the University of Lahore (UOL), Pakistan, under approval number UOL/IREB/25/08/0010. The study was mainly conducted at University of Lahore Teaching Hospital (ULTH). Additional permission for data collection was also sought from the ULTH. Two tools were used in this study. The QAB-original, which is entirely and freely available on the official website of University of Queensland language neuroscience laboratory and is published under a Creative Commons Attribution (CC BY) license also presented as supporting information (S3 File QAB-English materials (ZIP)). The other tool was the final version of QAB-Urdu which was developed in this study and is presented as supporting information (S1 File QAB-Urdu (QAB-U) Materials (ZIP). Data collection took place from July 2025 to September 2025. A total of 64 participants were included in this study. 44 stroke patients and 20 healthy individuals took part in the validation and reliability phase. A purposive sampling technique was used to recruit the participants throughout the study. The stroke patients were recruited from rehabilitation settings at ULTH, Pakistan and were medically stable at the time of assessment. The sample size for stroke patients was calculated using G*Power 3.1 software [15]. Sample size was calculated using the following parameters: effect size (r) = 0.40 (medium effect) [16], α error probability = 0.05, power (1 – β) = 0.80. The expected correlation coefficient used for planning was guided by earlier QAB validation studies, which reported moderate to strong correlations between adapted and original versions [17]. Inclusion criteria for these participants were: aged 18 years or older; diagnosed with stroke with aphasia or without aphasia; medically and cognitively stable at the time of testing; possessing sufficient attention and comprehension to complete assessment tasks; and bilingual in Urdu and English. The flow of these participants across the different phases of validation and reliability analyses, including final sample sizes contributing to each analysis, is summarized in Fig 1. Additionally, recruitment sources, inclusion and exclusion criteria of participants are also briefly explained under relevant subheadings of methodological section. Informed consent was obtained in written form from all the participants in each phase. In cases where participants with stroke were unable to complete the written consent form due to hemiparesis or hemiplegia, informed consent was obtained through their primary caregivers, who completed the form on their behalf. Participants were well informed regarding the aim of the study prior to data collection. Data collected in each phase was analysed using SPSS version 31.

### Phase 1: Translation and Adaptation Phase

This phase followed Brislin’s forward and back-translation model for translation and adaptation of the original QAB [18]

**Forward translation:** Three subject-matter experts (Assistant Professors with 5 + years of experience in dealing with aphasia) participated in the forward translation of the QAB into Urdu. Experts were selected to ensure bilingual proficiency (English and Urdu) and in-depth knowledge of aphasia and clinical assessment tools. The experts were asked to translate each item of the QAB from English to Urdu with conceptual accuracy, maintaining the original meaning and clinical intent. They were instructed not to omit any item, but to suggest culturally appropriate alternatives where necessary.

**Expert panel:** Three experts participated in the consensus meeting. All were assistant professors (doctoral scholars) and clinicians specialized in speech and language pathology. Each translated item of the Urdu version of the QAB was reviewed in a formal consensus meeting for conceptual accuracy, clinical relevance, and linguistic clarity.

**Back translation:** Three other PhD scholars in speech-language pathology who were proficient in Urdu and English participated in the back translation process. The finalised Urdu version of the QAB was independently translated back into English by the three selected scholars. The translators were given the same instructions as those in the forward translation phase, focusing on conceptual fidelity and linguistic accuracy rather than literal translation.

**Expert panel:** The same three experts from the forward translation panel participated in reviewing the back-translated version. The expert panel compared the back-translated English version of the QAB-Urdu with the original English items. They carefully examined each item to ensure conceptual consistency and linguistic accuracy.

**Pilot testing:** A total of 20 healthy individuals were included in the pilot testing phase. The chosen number aligned with methodological recommendations suggesting that 20–30 participants are sufficient for pilot studies [19]. Inclusion criteria for these participants were healthy individuals with no history of neurological or psychiatric illness, bilingual proficiency in Urdu and English, an age range of 20–30 years, and willingness to participate and provide feedback. Exclusion criteria included individuals with any history of stroke, head injury, diagnosed language disorder, and hearing or visual impairment that could affect performance. This testing was conducted in a quiet clinical environment at the ULTH to ensure standardized administration conditions. Each participant was individually administered the Urdu version of the QAB by trained Speech-Language Pathologists. The aim was to evaluate clarity of instructions, comprehensibility of pictorial stimuli, and cultural appropriateness. Participants were also asked to provide feedback on instructions and picture recognisability, while administration time was recorded to assess feasibility in clinical practice.

### Phase 2: Validity of QAB-Urdu

**Content validity ratio (CVR) of the QAB-Urdu version:** A total of four expert speech- language pathologists participated in the content validation process. Each expert was affiliated with the Department of Rehabilitation Sciences, UOL, and was highly experienced in the assessment and treatment of aphasia. The selection was based on their proficiency in both English and Urdu, as well as their clinical experience with aphasia assessment and intervention. Each expert was provided with a content validation form developed from the Urdu version of the QAB. The battery consisted of 57 items per form across three forms (Form 1, 2, and 3). Experts rated every single item using a three-point scale based on [20]: (1) essential, (2) useful but not essential, (3) not necessary. Experts were specifically instructed to evaluate each item for clinical relevance for Urdu-speaking individuals with aphasia, as well as for its semantic, syntactic, and pragmatic equivalence with the original English items. The CVR for each item was calculated using the following formula: $CVR=ne-(N/\text{2})/(\frac{N}{\text{2}})$, where n_e_ = number of experts who rated the item as ‘essential’ and N = total number of experts. Content validity indices were reported separately for Form 1, 2, and 3. The Item-Content Validity Index (I-CVI) for each item was calculated as the proportion of experts rating the item as essential (I-CVI = n_e_/N). The Scale-Content Validity Index (S-CVI) was calculated using the average method (S-CVI/Ave), defined as the mean of I-CVI values across all 57 items within each form.

**Linguistic equivalence and agreement analysis:** In this phase, the Urdu and English versions of the QAB were administered to 44 stroke patients to examine cross-language comparability. All participants involved in this phase were bilingual in Urdu and English, based on clinical history and self-report obtained during recruitment. Formal assessment of bilingual proficiency or language dominance was not conducted. The aim of this phase was to examine functional comparability between English and Urdu versions within the same individuals; hence, a within-subject design was employed, allowing each participant to serve as their own control. Each participant was administered both the English and Urdu versions of QAB. To minimize practice and memory effects, the two versions were administered with an interval of 5–7 days, and the order of administration was counterbalanced across participants to control for potential order effects. Scores for each subtest (Word Comprehension, Sentence Comprehension, Word Finding, Grammatical Construction, Motor Speech, Repetition, and Reading Aloud), as well as the overall QAB score, were calculated using the scoring system of the original QAB [13] and were then documented for both versions. The data were then analysed to evaluate functional comparability across versions. Given the cultural and contextual modifications added during adaptation, this analysis aimed to assess consistency and agreement between versions rather than strict item-level equivalence. Wilcoxon signed-rank tests were used to examine within-subject differences across subtests, Pearson correlation coefficient was used to assess the strength of association between scores, and Intraclass Correlation Coefficients (ICC; two-way mixed-effects model, absolute agreement) were used to evaluate agreement between Urdu and English versions.

**Construct validity (known-group approach):** Construct validity of the Urdu version of the QAB was examined using a known-group approach, in which participants with and without clinically diagnosed aphasia were included to assess the ability of QAB-Urdu to distinguish between groups expected to differ in language performance. This approach is consistent with early-stage validation studies that aim to provide evidence of construct validity with the help of group differentiation. A total of 44 participants with stroke were classified into two groups: Group 1 (aphasia) and Group 2 (non-aphasia), based on their clinical diagnosis of aphasia documented in hospital medical records. This diagnosis had been established by neurologists as a part of clinical evaluation, primarily based on the area and extent of brain lesions. For statistical analysis, diagnostic status was treated as a binary variable (aphasia = 1, no aphasia = 0). Diagnostic status was used as a grouping variable for known-groups comparison and as the reference standard for Receiver Operating Characteristic (ROC) analysis. Before statistical testing, the distribution of QAB overall scores was assessed for normality using the Shapiro-Wilk test, supplemented by visual inspection of box plots. Normality testing is essential to decide whether parametric or nonparametric methods are appropriate. Since the distributions were significantly non-normal in both groups, the nonparametric Mann-Whitney U test was used to compare the two groups on the score of the overall QAB and each subtest. In addition, descriptive statistics (median and interquartile range [IQR]) were calculated to describe the central tendency and variability of scores for both groups.

**Preliminary receiver operating characteristic (ROC):** Preliminary ROC analysis was conducted to evaluate the diagnostic performance of the QAB-Urdu for differentiating between participants with and without clinically diagnosed aphasia. The provisional cutoff score was determined using Youden’s Index. This was considered a preliminary ROC analysis because it was conducted on a relatively small sample (n = 44), including a limited number of aphasia cases.

### Phase 3: Reliability of QAB-Urdu

**Inter-rater reliability:** To evaluate inter-rater reliability of the QAB-Urdu, a subsample of 20 participants with stroke was independently scored by two trained Speech-Language Pathologists (Rater 1 and Rater 2) after an interval of 2 hours. Inter-rater reliability was assessed using two complementary statistical approaches: (1) Pearson’s correlation coefficient to measure the association between raters’ scores, and (2) ICC (two-way mixed-effects model, absolute agreement) to determine the level of absolute agreement. This multi-method approach was chosen because each statistic provides different but complementary information. Pearson’s r measures association and ICC reflect absolute agreement between raters.

**Test-retest reliability:** Test-retest reliability of the QAB-Urdu was assessed to examine the stability of scores over time. A subset of participants (n = 20) with stroke was re-administered the same QAB Form 1 after a two-week interval. The test-retest reliability was evaluated for each subtest and for the overall QAB score. Both Pearson’s correlation and ICC (two-way mixed-effects model, absolute agreement, single-measure) were calculated. Single-measure ICCs were reported as they reflect the reliability of a single administration of QAB-Urdu at each testing interval. Correlation coefficients above 0.70 and ICC values above 0.75 were considered indicative of acceptable reliability, while values above 0.90 were considered excellent.

**Internal consistency:** Internal consistency of the QAB-Urdu was examined using Cronbach’s alpha coefficient. The analysis was conducted on the seven subtests (Word Comprehension, Sentence Comprehension, Word Finding, Grammatical Construction, Motor Speech, Repetition, and Reading), which together constitute the overall score of QAB. Cronbach’s α values ≥ 0.70 were considered acceptable, ≥ 0.80 good, and ≥ 0.90 excellent. Additionally, corrected item-total correlations and ‘alpha if item deleted’ statistics were calculated to assess the contribution of each subtest to the overall scale.

**Parallel forms reliability:** The three forms of QAB-Urdu were adapted to be structurally comparable, with equivalent distribution of subtests and item types across all forms, following the structure of the original QAB. To evaluate the consistency of performance across the three forms of the QAB-Urdu, a subsample of 10 participants with stroke was used. All the participants in this subsample were administered Forms 1, 2, and 3, and the order of administration was counterbalanced to minimize order effects. Two statistical procedures were applied: (1) the Friedman test, a non-parametric test for repeated measures, was used to compare the total scores across the three forms to check if there were significant differences in performance, (2) Spearman’s rank correlation was used to examine the strength of association between the total scores of the three forms, thereby assessing the consistency of performance across them. However, because of a small number of participants, these analyses were intended to provide preliminary evidence of consistency across forms.

## Results

### Phase 1: Translation and Adaptation of QAB-Urdu

During the forward translation, multiple lexical and visual modifications were proposed across all three forms to enhance familiarity and cultural relevance. The major adjustments were concentrated in the subtest of word comprehension and picture naming. For example, items such as “violin,” “saxophone,” and “piano” were replaced with culturally recognizable equivalents like “kulhara, rasi, and hatori”. Similarly, expression such as “how you met your husband/wife?” was adapted to “your first job in life” to maintain cultural and religious appropriateness.

Following the expert review, 32 items were refined to better reflect linguistic naturalness, contextual familiarity, and phonemic association. Adjustments included substitution of musical references with locally meaningful items, revision of unfamiliar animals and object names, and phrasing of sentence stimuli for natural Urdu syntax. Examples include “pig” changed to “monkey” for cultural acceptability, “pyramid” was replaced by “mountain,” and “boy is kissing the girl” adapted to “boy is waking up the girl.” The expert panel confirmed that these changes retain clinical intent while improving recognition and reducing ambiguity.

Back translation demonstrated that the Urdu version maintained semantic and conceptual fidelity with the original English QAB. After the final expert verification, all items were reorganized following the same sequence as the original tool. Visual stimulus cards were redesigned with clearer and more culturally familiar images to enhance comprehension, in collaboration with professional graphic designers.

Pilot testing revealed that the Urdu version was easily understood and could be administered within 15–20 minutes, consistent with the original QAB time frame. Participants completed all tasks with minimal difficulty, though few images required substitution for improved recognizability. These refinements improved face validity and usability without altering the underlying psycholinguistic structure.

Overall, the adaptation resulted in the QAB-Urdu version that preserved the main clinical intent of the original tool while incorporating culturally and linguistically relevant modifications. Given that some changes extended beyond direct lexical translation to include contextual and pragmatic adjustments, the Urdu version should be considered a culturally adapted and modified version rather than a strictly equivalent version of the original tool.

### Demographic characteristics of participants

Demographics of total number of participants with stroke involved in different steps of validation and reliability phases are shown in Table 1. Detailed clinical variables such as time since stroke onset, lesion location, stroke type, and current language therapy status were not systematically recorded for all participants and therefore were not included.

**Table 1:** Demographic Characteristics of Participants (n = 44).

Variable	Category	Frequency (n)	Percentage (%)
Age Range	35–44	3	6.8
	45–54	5	11.4
	55–74	33	75.0
	75–80	3	6.8
Gender	Male	29	65.9
	Female	15	34.1
Education	Primary education	19	43.2
	Secondary	20	45.4
	Graduate	4	9.0
	Postgraduate	1	2.2
Medical Diagnosis	Stroke with Aphasia	7	15.9
	Stroke without Aphasia	37	84.1
Handedness	Right-Handed	39	88.6
	Left-Handed	5	11.4

### Phase 2: Validation of QAB-Urdu

**CVR of QAB-Urdu:** The I-CVI scores for the adapted Urdu version of QAB ranged from 0.75 to 1.00 across all three forms, as shown in Table 2.

**Table 2:** Summary of I-CVI and S-CVI for QAB-Urdu Forms.

Form	Total Items	Items with I-CVI = 1.00	Items with I-CVI = 0.75	S-CVI/Ave
Form 1	57	54	3	0.98
Form 2	57	57	0	1.00
Form 3	57	53	4	0.98

I-CVI, Item-Content Validity Index; S-CVI, Scale-Content Validity Index; Ave, Average.

**Linguistic equivalence and agreement analysis:** Results of the Wilcoxon signed-rank test showed that only word comprehension, reading, and the overall QAB score had p-values below the significance threshold, while all other subtests showed p > 0.05, indicating no statistically significant differences between the Urdu and English versions in all those areas as shown in Table 3. Pearson’s correlation coefficients indicated moderate to strong associations between the Urdu and English versions across subtests, reflecting consistency in performance patterns. On the other hand, ICC values showed moderate to high agreement between the two versions, while acknowledging that the Urdu version represents a culturally adapted form of the original version. The correlation coefficient shows the strength of association between scores, whereas ICC values provide a measure of absolute agreement.

**Table 3:** Linguistic Correlation of English and QAB-Urdu Scores (n = 44).

Subtest	Wilcoxon signed-rank test	Pearson r	ICC (Average Measures)	95% CI
Word Comprehension	0.048	0.60	0.75	0.62–0.82
Sentence Comprehension	0.062	0.82	0.89	0.81–0.94
Word Finding	0.052	0.62	0.77	0.64–0.83
Grammar Construction	0.070	0.81	0.88	0.80–0.93
Motor Speech	0.059	0.80	0.87	0.79–0.92
Repetition	0.065	0.79	0.86	0.78–0.91
Reading	0.041	0.83	0.90	0.82–0.94
QAB Overall	0.050	0.80	0.86	0.79–0.91

ICC, Intraclass Correlation Coefficient; CI, Confidence Interval.

**Fig 2:**
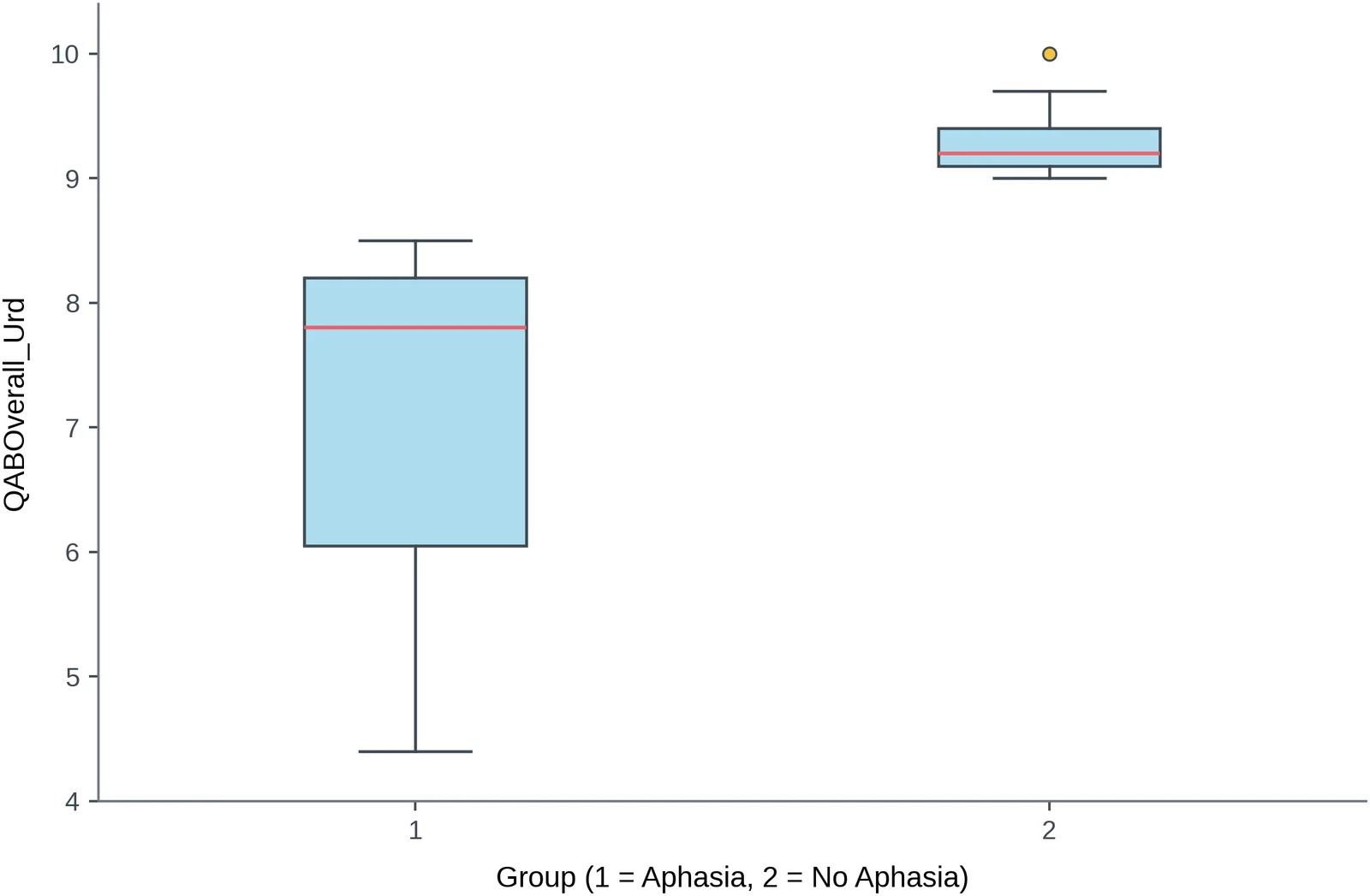
Boxplot of QAB Overall Urdu Scores.

**Construct validity:** Participants were categorized based on their clinical diagnosis into aphasia (n = 7) and non-aphasia (n = 37) groups, and this diagnostic classification was used as the reference standard for subsequent analysis. Normality testing was conducted separately for the aphasia and non-aphasia groups. The Shapiro-Wilk test was statistically significant as shown in Table 4, indicating the assumption of normality was violated for the QAB overall scores in both groups. Specifically, the aphasia group (n = 7) demonstrated a Shapiro-Wilk statistic of 0.739 (p < 0.001), and the non-aphasia group (n = 37) showed a Shapiro-Wilk statistic of 0.879 (p < 0.001). These results suggested that the data were not normally distributed. Visual inspection (box plot) provided further evidence of non-normality. The boxplot (Fig 2) also highlighted clear group separation, with the non-aphasia group achieving higher scores and displaying less variability compared to the aphasia group, which showed lower scores and greater dispersion.

**Table 4:** Test of Normality for QAB Overall Score.

Group	Test	Statistic	df	p-value
Aphasia (n = 7)	Shapiro-Wilk	0.739	7	< 0.001
Non-Aphasia (n = 37)	Shapiro-Wilk	0.879	37	< 0.001

df, degrees of freedom.

Given the violation of normality, Mann-Whitney U test were performed to compare the aphasia and non-aphasia groups on the QAB overall score and all subtests. The results consistently demonstrated statistically significant differences between groups (p < 0.05) with the non-aphasia group performing better across all domains. The strongest group differentiation was observed for the overall QAB score (U = 0.00, Z = −4.193, p < 0.001), indicating strong separation between the two groups. These results are demonstrated in Table 5.

**Table 5:** Mann-Whitney U Test Results for QAB Overall and Subtests.

Subtest	Mann–Whitney U	Test Statistic	p-value
Word Comprehension	11.50	−3.799	< 0.001
Sentence Comprehension	5.00	−4.002	< 0.001
Word Finding	30.00	−3.201	< 0.001
Grammar Construction	23.50	−3.411	< 0.001
Motor Speech	25.00	−3.363	< 0.001
Repetition	4.00	−4.036	< 0.001
Reading	3.50	−4.053	< 0.001
**QAB Overall**	**0.00**	−4.193	< 0.001

To complement inferential tests, descriptive statistics, as shown in Table 6 were calculated for each subtest and the overall score. The non-aphasia group consistently had higher median scores with narrower interquartile ranges, reflecting greater homogeneity in language abilities. Conversely, the aphasia group had lower scores with wider interquartile ranges, reflecting greater heterogeneity and variability in performance.

**Table 6:** Descriptive Statistics of QAB-U Subtests and Overall Scores.

Subtest	Group	Median (IQR)
Word Comprehension	Aphasia	7.8 (2.5)
	Non-Aphasia	9.3 (0.8)
Sentence Comprehension	Aphasia	7.8 (3.6)
	Non-Aphasia	9.2 (0.7)
Word Finding	Aphasia	8.3 (3.8)
	Non-Aphasia	9.3 (0.8)
Grammar Construction	Aphasia	8.3 (3.8)
	Non-Aphasia	9.3 (0.8)
Motor Speech	Aphasia	8.2 (4.0)
	Non-Aphasia	9.2 (0.8)
Repetition	Aphasia	8.2 (4.0)
	Non-Aphasia	9.2 (0.8)
Reading	Aphasia	7.3 (2.4)
	Non-Aphasia	9.2 (0.8)
QAB Overall	Aphasia	7.8 (3.7)
	Non-Aphasia	9.2 (0.3)

SD, Standard Deviation; IQR, Interquartile range.

These findings suggest that the QAB-Urdu can discriminate between stroke patients with and without clinically diagnosed aphasia, supporting its known-group validity.

**Fig 3:**
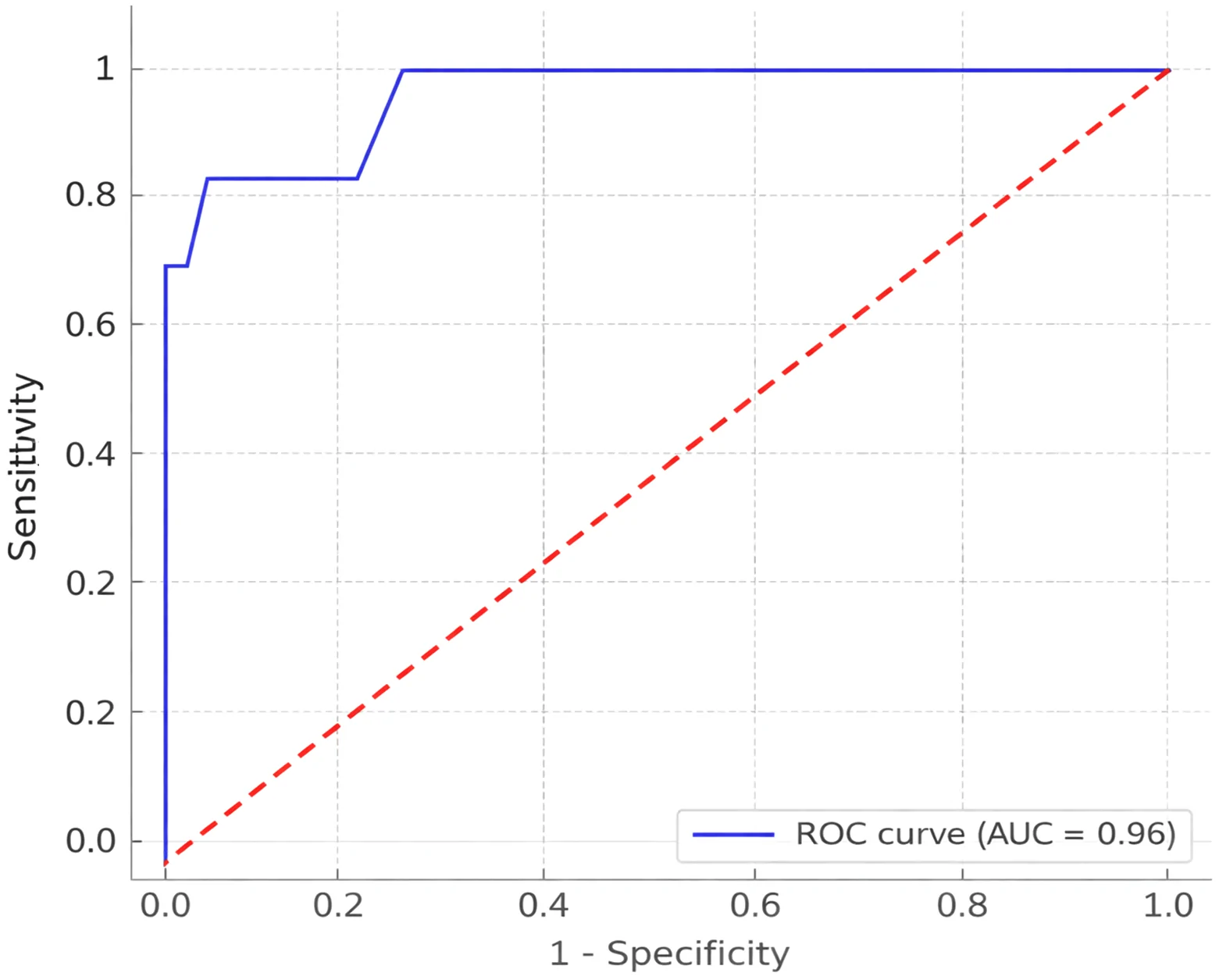
ROC Curve of the Overall QAB-Urdu.

**Preliminary ROC analysis:** The area under the curve (AUC), as visible in Fig 3 was 0.96 (SE = 0.03, 95% CI: 0.90–0.99, p < 0.001), indicating good diagnostic performance. Using a provisional cutoff value of 8.9, the overall QAB-Urdu demonstrated a sensitivity of 86% and a specificity of 92% in the present sample, suggesting that most aphasia cases were correctly identified and most non-aphasia cases were correctly excluded in this sample. These results are also visible in Table 7. Although the AUC estimate is high, this finding must be interpreted with caution, due to relatively a small number of aphasia cases (n = 7) included in this study, which may limit the stability of diagnostic estimates.

**Table 7:** Preliminary ROC Analysis for QAB-Urdu (n = 44).

Variable	AUC	SE	95% CI	p-value	Cutoff	Sensitivity	Specificity
QAB-Urdu Overall	0.96	0.03	0.90–0.99	< 0.001	8.9	86%	92%

AUC, Area Under Curve; CI, Confidence Interval.

### Phase 3: Reliability of QAB-Urdu

**Inter-rater reliability**: For all subtests, Pearson correlation coefficients were statistically significant (p < 0.001), reflecting the strength of association between raters’ scores, while ICC analysis further supported these findings, with all subtests and the overall score showing ICC values above the accepted threshold of 0.75 [21], demonstrating excellent inter-rater reliability across individual subtests as well as the composite QAB-Urdu score (Table 8).

**Table 8:** Inter-rater reliability of QAB-Urdu.

Subtest	R1 Mean (SD)	R2 Mean (SD)	Pearson r (p)	ICC average measure (95% CI)
Word Comprehension	8.70 (0.58)	8.72 (0.55)	0.794, p < 0.001	0.889 (0.719–0.956)
Sentence Comprehension	8.52 (0.68)	8.65 (0.73)	0.840, p < 0.001	0.906 (0.765–0.963)
Word Finding	8.48 (0.57)	8.40 (0.66)	0.897, p < 0.001	0.938 (0.847–0.975)
Grammar Construction	8.57 (0.89)	8.72 (0.92)	0.890, p < 0.001	0.937 (0.842–0.975)
Motor Speech	8.38 (0.62)	8.36 (0.75)	0.776, p < 0.001	0.871 (0.670–0.949)
Repetition	8.32 (0.79)	8.35 (0.96)	0.912, p < 0.001	0.946 (0.864–0.979)
Reading	8.51 (0.73)	8.38 (0.67)	0.801, p < 0.001	0.883 (0.710–0.953)
**QAB-Urdu Overall**	8.50 (0.27)	8.51 (0.29)	0.921, p < 0.001	0.960 (0.902-0.984)

SD, Standard Deviation; ICC, Intraclass Correlation Coefficient; CI, Confidence Interval.

**Parallel forms reliability:** Results presented in Table 9 showed that the Friedman test revealed no statistically significant difference among the three forms χ² = 1.285, p = 0.526, indicating that participants’ performance did not differ systematically across versions.

**Table 9:** Difference analysis for QAB-Urdu Forms 1,2 and 3.

Measure	χ²	p
QAB-Urdu Form 1,2 and 3	1.285	0.526

Correlation analysis, as shown in Table 10, reflected statistically significant positive associations between the total scores of all forms (r = 0.982–0.995, p < 0.001), indicating a high degree of consistency in performance across forms. Correlation, however, shows association rather than the agreement. However, it should be noted that lack of significant differences does not confirm equivalence, and such findings should be interpreted as preliminary evidence of consistency rather than definitive equivalence.

**Table 10:** Spearman correlations between QAB-Urdu Forms (n = 10).

Form Comparison	Spearman r	p-value
Form 1 × Form 2	0.995	< 0.001
Form 1 × Form 3	0.982	< 0.001
Form 2 × Form 3	0.986	< 0.001

**Test-retest reliability:** Results of the test-retest analysis, showed in Table 11 demonstrated stability across all QAB subtests and the overall score. Pearson correlations ranged from 0.895 (Reading) to 0.985 (Word Comprehension), all statistically significant at p < 0.01. The ICC values also ranged from 0.894 (Reading) to 0.984 (Word Comprehension) for single measures, indicating stable agreement across test-retest administration.

**Table 11:** Test- Retest Reliability of QAB-Urdu Subtests and Overall Score (n = 20).

Subtest	Pearson Correlation	ICC (Single Measures)	95% CI
Word Comprehension	0.985	0.984	0.971–0.991
Sentence Comprehension	0.909	0.910	0.842–0.950
Word Finding	0.965	0.965	0.936–0.981
Grammatical Construction	0.934	0.935	0.884–0.964
Motor Speech	0.980	0.980	0.964–0.989
Repetition	0.984	0.984	0.970–0.991
Reading	0.895	0.894	0.815–0.941
QAB Overall	0.976	0.976	0.957–0.987

ICC, Intraclass Correlation Coefficient (single measure); CI, Confidence Interval.

**Internal consistency:** Cronbach’s alpha for the overall QAB-Urdu was 0.960, supporting internal consistency as shown in Table 12. Alpha values remained stable when any single subtest was deleted (ranging from 0.956 to 0.959**),** recommending that no individual subtest disproportionately affected the overall reliability. Additionally, the corrected item-total correlations for all subtests fell within an acceptable range (0.69–0.89**),** suggesting that each component contributed to the overall construct of the QAB.

**Table 12:** Internal Consistency of QAB-Urdu.

Subtest	Corrected Item–Total Correlation	Cronbach’s Alpha if Item Deleted
Word Comprehension	0.82–0.86	0.958
Sentence Comprehension	0.79–0.87	0.957
Word Finding	0.80–0.87	0.957
Grammatical Construction	0.82–0.89	0.956
Motor Speech	0.77–0.89	0.957
Repetition	0.79–0.85	0.957
Reading	0.69–0.83	0.959
Overall Scale	—	__

## Discussion

The present study aimed to translate, culturally adapt, and determine the psychometric properties of the Quick Aphasia Battery for Urdu-speaking stroke patients in Pakistan. Overall, QAB-Urdu demonstrated preliminary evidence of validity and reliability, with results comparable to previously reported findings from the Chinese [17], and Turkish [14] adaptations, and the original English version [13].

The adaptation process followed internationally recognized guidelines and previous QAB validation steps, emphasizing expert feedback, linguistic clarity, and pilot testing to ensure accuracy and cultural relevance. Following expert review, 32 items were refined to enhance linguistic naturalness, contextual familiarity, and phonemic appropriateness, reflecting the extent of adaptation required for the target population. This level of modification is comparable to that reported in Chinese adaptation, which also involved several item-level adjustments to align with cultural and linguistic norms. Like Turkish and Chinese versions, the Urdu adaptation demonstrated high content validity, ensuring that translation alone may be insufficient and adaptation is essential for diagnostic accuracy [14,17]. However, unlike strict linguistic equivalence, various modifications in the Urdu version involved contextual and pragmatic adjustments, for example, changes in culturally sensitive items and pictorial stimuli. Therefore, the QAB-Urdu should be considered a culturally adapted and modified version of the original tool rather than a strictly equivalent translation. Comparable approaches were also reported by a Pakistani Speech-Language Pathologist [22] during the development of UAAT, which highlighted the importance of expert-driven refinements for culturally appropriate assessment and content validity ratio for ensuring item appropriateness.

Linguistic equivalence and agreement analysis showed strong consistency in scores between the Urdu and English versions, as reflected by Pearson correlation values across subtests, along with moderate to high agreement, as indicated by ICC values. This provides additional evidence beyond previous QAB adaptations, including the original QAB and its Chinese and Turkish versions, which primarily focused on content and construct validity, by providing additional evidence of cross-language agreement between the original and adapted versions.

Construct validity in the current study was examined using a known-group validity approach, with significant group differences (p < 0.001) between aphasic and non-aphasic participants across all subtests, consistent with findings from the Chinese QAB [17] and the Swedish adaptation of the CAT [23]. These results indicate that the QAB-Urdu was able to discriminate between individuals with and without clinically diagnosed aphasia. However, known-group validity reflects only one aspect of construct validity and does not fully capture the underlying construct structure. While this approach provides beneficial evidence, the imbalance between groups should be considered when interpreting diagnostic performance. Another step followed in the Chinese [17] study for ensuring validity was discriminant validity. It was done by comparing WAB and C-QAB. Such comparison was not made in the current study due to the unavailability of standardised tools in Pakistani region.

Reliability outcomes of the QAB-Urdu were also strong. Inter-rater reliability showed consistent scoring, with no significant difference between raters, supported by significant correlations and high ICC values, aligning with the Chinese adaptation [17], which reported excellent inter-rater reliability (ICC = 0.998). Parallel form reliability also exhibited no significant difference across forms (p = 0.526), and strong correlations (r = 0.982–0.995), consistent with the Turkish adaptation [14], which reported similarly high correlations (r = 0.984–0.997) and no significant differences between forms (p = 0.538). These findings show a high degree of consistency in performance across forms. Yet, correlation reflects association rather than agreement, particularly given the small sample size (n = 10) and in the absence of additional agreement-based statistics, these results should be interpreted as preliminary evidence of consistency rather than definitive equivalence between forms. The overall internal consistency (Cronbach’s α = 0.96) and ICC values (0.87–0.95) were comparable to those reported in the Turkish study (α = 0.95; ICC = 0.94) [22] and Chinese study (α = 0.962; ICC = 0.998) [17], indicating strong measurement stability. While extremely high alpha values may sometimes indicate potential redundancy, corrected item – total correlation (0.69–0.89) and the stable values of alpha following item deletion reflect that all subtests contributed meaningfully to the construct of the scale without any evidence of problematic redundancy. These findings also align with the UAAT [22], which reported similar Cronbach’s alpha values, supporting the reliability of aphasia tools when culturally adapted.

The diagnostic accuracy of the QAB-Urdu, as indicated by the preliminary ROC analysis (AUC = 0.96, sensitivity = 86%, specificity = 92%), was consistent with that of the Chinese QAB (AUC = 0.99) [17] and the Swedish CAT-S (AUC = 0.97) [23], both demonstrating good discriminative ability. However, these findings should be interpreted with caution due to the relatively small number of aphasia cases (n = 7). Therefore, the identified cutoff should be interpreted as provisional, and further validation in larger samples is required before establishing definitive diagnostic thresholds. All the findings should be interpreted in the light of methodological limitations.

### Limitations

Various limitations of this study should be acknowledged. Firstly, relatively small sample size, specifically the number of participants with aphasia (n = 7) compared to stroke participants without aphasia, may affect the stability and generalizability of the findings. This imbalance may also affect the estimates of diagnostic performance, and it should be considered carefully while comprehending the results. Aphasia classification was made based on neurologists’ clinical evaluation and area of brain lesion; an independent standardized aphasia assessment was not used as a reference standard. This limitation should be considered when interpreting the known-group validity and diagnostic accuracy findings.

Participants were selected using a purposive sampling technique, which may introduce selection bias and limit the generalizability of the findings to broader populations. Future studies using a probabilistic sampling technique may enhance external validity. Moreover, several detailed clinical variables such as time post-stroke, location of lesion, type of stroke, and severity of impairment were not systematically recorded. Such factors may impact language performance and should be incorporated in future research to provide a more comprehensive clinical characterization. Additionally, even though participants were bilingual in English and Urdu, formal assessment of language proficiency and dominance was not evaluated. Variations in bilingual proficiency may have affected cross-language performance, but the use of a within-subject design partially mitigated inter-individual variability.

Pilot testing was conducted on younger healthy participants, which may not fully reflect the older clinical population included in the validation phase. While the approach was appropriate for evaluating clarity and feasibility, future studies should consider age-matched pilot samples to enhance validity. Also, construct validity was evaluated using known-group comparisons only. Although this approach is appropriate, additional psychometric evaluation such as convergent validity, exploratory or confirmatory factor analysis is suggested to establish structural validity more comprehensively. Moreover, parallel form reliability was evaluated using a subsample of 10 participants, which limits the generalizability of these findings. In addition, equivalence between forms was assessed using group comparison and correlation methods, whereas methods such as ICC were not calculated. Further studies should involve a larger number of participants and conduct agreement-based analyses to establish equivalence between forms. Finally, despite the ROC analysis reflecting discriminative ability, it should be considered preliminary due to a limited number of aphasia cases. The proposed cut-off values require validation in diverse and larger clinical samples before being applied in clinical practice. Future research should focus on including larger and more clinically heterogeneous samples, incorporating detailed clinical and linguistic profiles, and further evaluating the diagnostic and structural validity of the QAB-Urdu.

## Conclusion

The QAB-Urdu reflected preliminary validity and reliability for evaluating language performance in Urdu-speaking stroke patients. These findings show that the tool is culturally appropriate, internally consistent, and can distinguish between individuals with and without clinically diagnosed aphasia. The QAB-Urdu reflects the development of culturally adapted aphasia assessment tool for Urdu-speaking populations and has the potential to support both clinical practice and research in resource-limited settings.

## Supporting information

S1 File QAB-Urdu (QAB-U) Materials. This ZIP archive includes the complete Quick Aphasia Battery-Urdu developed in this study, including the translated scoring sheet, stimulus materials, and macro scoring sheet. All materials are openly available under a CC BY license. These materials are also hosted on the University of Queensland Language Neuroscience Laboratory website.
(ZIP)

S2 File Author permission. (PNG)

S3 File QAB-English Materials. This ZIP archive includes the complete Quick Aphasia Battery original version in English, including the scoring sheet, stimulus materials, and macro scoring sheet. All materials are openly available under a CC BY license.
(ZIP)
