# Supplementary material for: Adaptation of the Quick Aphasia Battery into Urdu and establishment of its psychometric properties
Source: PLoS One. 2026 Sep 1;21(9):e0356908. doi: 10.1371/journal.pone.0356908 (PMC13533341; doi:10.1371/journal.pone.0356908)
Supplement: Supplementary file 1 [file pone.0356908.s001.zip › QAB-Urdu/ scoresheets 1,2 and 3┌⌐┘ê┘è┘ö┌⌐ ╪o ┘ü█î╪▓█î╪o ╪¿█î┘╣╪▒█î.pdf]

کوئک افیزیا بیٹری

فارم ا

شریک کندہ ____________ معالج ____________ وقت ____________

تاریخ ____________ مقام ____________

- ہر جُز کا جواب دینے کے لیے چھ سیکنڈ کا وقت دیں۔ تین سیکنڈ کے بعد جوابات کو تاخیر میں شمار کریں اور اس کے تین نمبر دیں۔ صرف پہلے چھ سیکنڈ میں دیے گئے جواب کو نمبر دیں، سوائے اس کے کہ جہاں جواب چھ سیکنڈ سے پہلے شروع کیا جائے اور پھر جاری رکھا جائے۔
- پہلے مکمل جواب کو نمبر دیں۔(یعنی ایسا جواب نہیں جس کا غلط آغاز ہو یا وہ نامکمل ہو)
- اگر مریض درخواست کرے یا معالج کو مریض کے غلط سُننے یا توجہ دینے میں کوتاہی کا شُبہ ہو تو زبانی طور پر پیش کردہ جُز کو ایک بار دہرایا جا سکتا ہے۔ اگر کوئی جُز دہرایا جاتا ہے تو چھ سیکنڈ کی گنتی کو دوبارہ شروع کریں۔
- ایسی ڈِس آرتھریا یا اپریکسیا کی غلطی کو نظر انداز کریں جو کسی بھی صوتیہ کی شناخت کو متاثر نہ کر رہی ہو، سوائے اس کے کہ جب گُفتاری حرکت کی درجہ بندی کی جا رہی ہو۔

۱) شعور کی سطح

الف) کیا مریض طبی طور پر اس قدر مستحکم ہے کہ اس سے رابطہ کیا جا سکتا ہے؟ [۰ (رکو)] [۴]

رابطہ کیا جا سکتا ہے؟ درجہ بندی درج ذیل ہے: ۴ =ہاں؛ ۰ = نہیں، نرسوں اور ڈاکٹروں وغیرہ سے فیصلے کی بنیاد پر۔

ب) کیا مریض کو بیدار کیا جا سکتا ہے؟ [۰(رکو)] [۱(رکو)] [۲] [۳] [۴]

بیدار کیا جا سکتا ہے؟ درجہ بندی درج ذیل ہے: ۴= پہلے سے بیدار ہے یا ہلکے محرک سے بیدار ہوتا ہے؛ ۳= درمیانی درجے کے محرک سے بیدار ہوتا ہے؛ ۲= بیدار ہوتا ہے، لیکن بیداری برقرار نہیں رکھتا؛ ۱ = کچھ رِدِ عمل دکھاتا ہے لیکن بیدار نہیں ہوتا؛ ۰= کوئی جواب نہیں۔

باشعور و باخبر درجہ بندی درج ذیل ہے: ۴= ایک قابل فہم درست جواب (کسی بھی تقریر یا زبان کی غلطیوں کو یہاں نظر انداز کیا جائے گا)؛ ۳= تینوں ہاں یا نہیں سوالات کے درست جوابات؛ ۲= ہاں سوال پر درست اور آدھے نہیں سوالات

پر درست جوابات؛ ۱ = سمجھنے اور جواب دینے کی کوشش کی مشابہ علامت؛ ۰ = جواب اور سوال کو سمجھنے کی کوئی علامت نہیں دکھاتا ہے یا کوئی جواب نہیں دیتا۔

ت) کیا آپ مجھے بتا سکتے ہیں کہ ہم اس وقت کہاں ہیں؟ ________________ [۰] [۱] [۲] [۳] [۴]

اگر کوئی جواب نہیں یا غلط جواب: کیا ہم لائبریری میں ہیں؟ [ہاں] [نہیں] [کوئی جواب نہیں]

کیا ہم کھیل کے میدان میں ہیں؟ [ہاں] [نہیں] [کوئی جواب نہیں]

کیا ہم [صحیح جگہ] میں ہیں؟ [ہاں] [نہیں] [کوئی جواب نہیں]

ٹ) یہ کون سا مہینہ ہے؟ ________________ [۰] [۱] [۲] [۳] [۴]

اگر کوئی جواب نہیں یا غلط جواب: کیا یہ [غلط مہینہ] ہے؟ [ہاں] [نہیں] [کوئی جواب نہیں]

کیا یہ [غلط مہینہ] ہے؟ [ہاں] [نہیں] [کوئی جواب نہیں]

کیا یہ [درست مہینہ] ہے؟ [ہاں] [نہیں] [کوئی جواب نہیں]

ث) آپ کی عمر کتنی ہے؟ ________________ [۰] [۱] [۲] [۳] [۴]

اگر کوئی جواب نہیں یا غلط جواب: کیا آپ [غلط عمر] کے ہیں؟ [ہاں] [نہیں] [کوئی جواب نہیں]

کیا آپ [درست عمر] کے ہیں؟ [ہاں] [نہیں] [کوئی جواب نہیں]

کیا آپ [غلط عمر] کے ہیں؟ [ہاں] [نہیں] [کوئی جواب نہیں]

احکامات کی پیروی کر سکتا ہے؟ درجہ بندی مندرجہ ذیل ہے: ۴ = زبانی حکم کی پیروی کرتا ہے؛ ۳ = عمل کی نقل کرتا ہے؛ ۱ = زبانی حکم پر عمل کرنے یا عمل کی نقل کرنے کی مشابہ کوشش کرتا ہے؛ ۰ = جواب، حکم کو سمجھنے کا کوئی نشان نہیں دکھاتا یا کوئی جواب نہیں دیتا۔ اگر حالات یا مریض کے عوامل حکم دیتے ہیں تو متبادل آسان حکم کو تبدیل کیا جا سکتا ہے، اس صورت میں انہیں نوٹ کریں۔

ج) آنکھیں بند کریں۔ ________________ [۰] [۱] [۳] [۴]

اگر بند نہ کریں تو: اپنی آنکھیں بند کریں اور نقل کرنے کا اشارہ کریں۔

چ) میرا ہاتھ دبائیں۔ ________________________ [۰][۱][۳][۴]

اگر نہ کریں تو: مریض کا ہاتھ دبائیں اور نقل کرنے کا اشارہ کریں۔

مجموعی تاثر: درجہ بندی درج ذیل ہے: ۴= ہاں؛ ۳= ہاں، مگر کچھ کمی پیشی (مثلاً توجہ میں وقفہ)؛ ۲= کبھی کبھی ؛ ۱= معمولی؛ ۰= بالکل نہیں۔

ح) کیا مریض بیدار رہ سکتا ہے، توجہ برقرار رکھ سکتا ہے اور احکامات کی پیروی کر سکتا ہے؟

[۰(رکو)] [۱(رکو)] [۲(رکو)] [۳] [۴]

۲) گفتگو میں تسلسل

کم از کم تین منٹ تک شریک کندہ کے ساتھ بات چیت کریں، ان میں سے ایک یا زیادہ گفتگو کے عنوانات پر جیسے:

- جب آپ کا پہلا بچہ ہوا؟
- جب آپ نے زندگی کی سب سے پہلی سواری خریدی؟
- آپ جہاں رہتے ہیں وہاں کے بارے میں آپ کو کیا پسند ہے؟
- ایک وقت جب آپ واقع ہی خوفزدہ یا شرمندہ یا ناراض تھے؟
- بہترین سفر جو آپ نے کیا ہے؟
- سب سے بُرا سفر جو آپ نے کیا ہے؟
- جب آپ کی شادی ہوئی؟
- بچپن میں آپ کی پسندیدہ چھٹی؟
- بچپن کی کوئی خوشگوار یاد؟
- آپ کی پہلی نوکری؟
- آپ کی بچپن کی سب سے بُری یاد؟

تحریکی صفحہ ا پیش کریں۔ پوچھیں "یہاں کیا ہو رہا ہے؟"

الف) لڑکا لڑکی کو دھکا دے رہا ہے۔ ________________________________

ب) لڑکی لڑکے کو پکڑ رہی ہے ____________________________________

درج ذیل پیمانوں کا استعمال کرتے ہوئے تسلسل والی گُفتار کی درجہ بندی کریں۔ تشخیص میں تیار کی گئی دیگر تسلسل والی گُفتار پر بھی غور کریں۔ ۴= معمول کے مطابق: کوئی غلطی موجود نہیں یا عام حد کے اندر؛ ۳= معمولی: قابل شناخت لیکن کبھی کبھار؛ ۲= درمیانہ: بار بار لیکن وسیع نہیں؛ ۱= نمایاں: وسیع لیکن ہر جگہ نہیں؛ ۰= شدید: زیادہ تر یا تمام الفاظ میں واضح۔

افیزک تسلسل والی گفتگو کی خصوصیات	۰	۱	۲	۳	۴
الفاظ کی لمبائی اور پیچیدگی میں کمی	واحد لفظ	گفتگو کی اوسط لمبائی: دو الفاظ	گفتگو کی اوسط لمبائی: تین سے چار الفاظ	گفتگو کی اوسط لمبائی: پانچ سے سات الفاظ	معمول کے مطابق
بول چال کی شرح میں کمی	صفر سے چوبیس لفظ فی منٹ	پچیس سے انچاس الفاظ فی منٹ	پچاس سے چوہتر الفاظ فی منٹ	پچھتر سے ننانوے الفاظ فی منٹ	سو سے زیادہ الفاظ
بے قاعدہ گرامر: الفاظ اور صرفیات کو چھوڑنا، خاص طور پر قواعدی الفاظ؛ "مختصر جملہ بندی"	شدید	نمایاں	درمیانہ	معمولی	معمول کے مطابق

غیر مربوط گرامر: قواعدی الفاظ کا متبادل؛ الفاظ اور جملوں کا نامناسب جوڑ؛ خراب نحو	شدید	نمایاں	درمیانہ	معمولی	معمول کے مطابق
لفظ یاد آنے میں مشکل: لفظ تلاش کرنے میں وقفہ، الفاظ کی تلاش میں ناکامی، ترک شدہ الفاظ، کامیاب اشارہ کنائی	شدید	نمایاں	درمیانہ	معمولی	معمول کے مطابق
غیر مرکزی گفتگو: مبہم الفاظ، قواعدی الفاظ کی کثرت، ناکام اشارہ کنائی، لفظوں کا غیر واضح معنی	شدید	نمایاں	درمیانہ	معمولی	معمول کے مطابق
معنوی لفظی تبدیلیاں: کھلے زمرے کے الفاظ کا متبادل، بار بار پچھلے لفظ کی دہرائی	شدید	نمایاں	درمیانہ	معمولی	معمول کے مطابق
صوتی لفظی تبدیلیاں اور خود ساختہ الفاظ	شدید	نمایاں	درمیانہ	معمولی	معمول کے مطابق
خود درستگی: غلط آغاز، روکی گئی یا دوبارہ شروع کی گئی جملہ بندی، خود اصلاحی کوشش	شدید	نمایاں	درمیانہ	معمولی	معمول کے مطابق

**مجموعی طور پر گفتگو میں خرابی**	گفتگو ٹکڑوں میں ہو: گفتگو قائم رکھنے کی مکمل ذمہ داری ممتحن پر ہو۔	محدود گفتگو ہو: شریک کندھا بھی گفتگو قائم کرنے کی ذمہ داری لے رہا ہو۔	روزمرہ کے موضوعات پر گفتگو کر سکتا ہو؛مگر اس سے باہر گفتگو محدود ہو۔	خرابی واضح ہو، مگر گفتگو تمام موضوعات پر کر سکتا ہو۔	کوئی خرابی واضح نہ ہو۔

اگر ممکن نہ ہو:

- کوئی خود ساختہ گفتگو نہیں
- صرف بے ربط بڑبڑاہٹ
- صرف بار بار دہرائے جانے والے مخصوص جملے یا الفاظ
- دس الفاظ فی منٹ سے کم، بنیادی طور پر صرف ہاں یا نہ، چند ایک الفاظ یا ہلکی سی کوشش

۳) لفظ کی سمجھ

تحریکی صفحہ ۲ پیش کریں۔ کہیں "مجھے دکھائیں"۔ درج بندی حسب ذیل ہے: ۴= درست؛ ۳= درست لیکن تین سیکنڈ کی تاخیر سے زیادہ، خوددرستگی یا بار بار دہرائی؛ ۱= متعلقہ ورق؛ ۰= متعلقہ متبادل ورق یا چھ سیکنڈ کے اندر کوئی جواب نہیں۔

الف) شیر متعلقہ: زرافہ، گھوڑا ________________ [۴][۳][۱][۰]

ب) پلیٹ متعلقہ: گلاس، چمچ ________________ [۴][۳][۱][۰]

پ) چمچ متعلقہ: پلیٹ، گلاس ________________ [۴][۳][۱][۰]

ت) زرافہ متعلقہ: شیر، گھوڑا ________________ [۴][۳][۱][۰]

تحریکی صفحہ ۳ پیش کریں۔

ٹ) لکڑی متعلقہ: مکڑی ________________ [۴][۳][۱][۰]

ث) لوٹا متعلقہ: طوطا ________________ [۴][۳][۱][۰]

ج) جوتا متعلقہ: طوطا ________________ [۴][۳][۱][۰]

چ) بکری متعلقہ: مکڑی ________________ [۴][۳][۱][۰]

۴) جملے کی سمجھ

کہیں "ہاں یا نہیں" میں جواب دیں۔ درجہ بندی حسب ذیل ہے: ۴ = درست سر ہلانا، ہاں یا نہیں کے دیگر سیاق و سباق کے مطابق مناسب متبادل جیسے" کبھی کبھی"، "مجھے ایسا نہیں لگتا"، یا غیر معمولی ردِ عمل جو سمجھ کا مظاہرہ کرتے ہیں قابل قبول ہیں؛ ۳= درست، لیکن، تین سیکنڈ سے زیادہ تاخیر، خود درستگی یا بار بار دہرائی؛ ۲= غیر معمولی ردِ عمل جہاں یہ واضح نہیں ہے کہ مریض سمجھتا ہے یا نہیں۔ ۱ = غلط، لیکن جواب کچھ جزوی فہم کی تجویز کرتا ہے؛ ۰= غلط یا چھ سیکنڈ کے اندر کوئی جواب نہیں۔

الف) کیا آپ ایک (مرد یا عورت) ہیں؟ (ہاں) ________ [۰][۱][۲][۳][۴]

ب) کیا میں ایک (مرد یا عورت) ہوں؟ (ہاں) ________ [۰][۱][۲][۳][۴]

پ) کیا آپ کلہاڑی سے گھاس کاٹتے ہیں؟ (نہیں) ________ [۰][۱][۲][۳][۴]

ت) کیا بچوں کی دیکھ بھال آیا کرتی ہے؟ (ہاں) ________ [۰][۱][۲][۳][۴]

ٹ) کیا آپ دروازہ چابی سے کھولتے ہیں؟ (ہاں) ________ [۰][۱][۲][۳][۴]

ث) اگر آپ جانے والے ہیں، تو کیا ابھی تک جا چکے ہیں؟ (نہیں) ________ [۰][۱][۲][۳][۴]

ج) کیا پولیس گواہوں سے پوچھ گیچھ کرتی ہے؟ (ہاں) ________ [۰][۱][۲][۳][۴]

چ) کیا مریض ڈاکٹروں کا علاج کرتے ہیں؟ (نہیں) ________ [۰][۱][۲][۳][۴]

ح) اگر میں پارک میں تھا، جب آپ پہنچے تو کیا میں وہاں پہلے سے موجود تھا؟ (ہاں) ________ [۰][۱][۲][۳][۴]

خ) اگر میں آپ کو بتاؤں کہ میں چائے پیتا تھا کیا آپ کو لگتا ہے کہ میں ابھی بھی چائے پیتا ہوں؟ (نہیں)

________ [۰][۱][۲][۳][۴]

د) اگر آپ سیڑھیاں چڑھنے والے ہیں تو کیا آپ ابھی بھی نیچے کھڑے ہیں؟ (ہاں) ________ [۰][۱][۲][۳][۴]

ڈ) کیا بلیوں کا تعاقب چوہے کرتے ہیں؟ (نہیں) ________ [۰][۱][۲][۳][۴]

۵) تصویر کو نام دینا

تحریکی صفحہ ۵ پیش کریں اور پوچھیں "یہ کیا ہے؟"۔ درجہ بندی حسب ذیل ہے: ۴= قابل قبول درست جواب؛ ۳= درست، لیکن تین سیکنڈ سے زیادہ تاخیر سے یا خود درست کیا ہو؛ ۲= کم از کم نِصف صوتیات درست ہوں یا ہدف پر اپریکزک غلطی؛ ۱= ہدف سے کچھ تعلق؛ ۰= غیر متعلقہ جواب یا چھ سیکنڈ کے اندر کوئی جواب نہیں۔

الف) کتا ______________________ [۰][۱][۲][۳][۴]

ب) پینسل ______________________ [۰][۱][۲][۳][۴]

پ) کرسی ______________________ [۰][۱][۲][۳][۴]

ت) سانپ ______________________ [۰][۱][۲][۳][۴]

ٹ) جھولا ______________________ [۰][۱][۲][۳][۴]

ث) سیڑھیاں ______________________ [۰][۱][۲][۳][۴]

٦) دہرائی

کہیں "میرے بعد دہرائیں"۔ درجہ بندی حسب ذیل ہے: ۴= درست، (ث) مختصر شکل کو مکمل میں تبدیل کر دیں؛ ۳= درست، لیکن تین سیکنڈ سے زیادہ تاخیر سے، خود درست کیا ہو یا بار بار دہرایا ہو؛ ۲= (الف سے ت) کم از کم نِصف صوتیات درست ہوں یا ہدف پر اپریکزک غلطی، (ٹ اور ث) میں کم از کم نِصف الفاظ ہوں؛ ۱= ہدف سے کچھ تعلق، ٹ اور ث کے لیے دو سے زیادہ الفاظ ہوں؛ ۰= غیر متعلقہ جواب یا چھ سیکنڈ کے اندر کوئی جواب نہیں۔

الف) ہنسی ______________________ [۰][۱][۲][۳][۴]

ب) برخوردار ______________________ [۰][۱][۲][۳][۴]

پ) قینچی ______________________ [۰][۱][۲][۳][۴]

ت) اندیشہ ______________________ [۰][۱][۲][۳][۴]

ٹ) سورج مشرق سے نکلتا ہے۔ ______________________ [۰][۱][۲][۳][۴]

ث) پُرعزم صحافی نے دریافت کیا کہ ہم کہاں جا رہے تھے۔ ______________________ [۰][۱][۲][۳][۴]

۷) بلند آواز سے پڑھنا

تحریکی صفحہ ٦ پیش کریں۔ کہیں "ان الفاظ اور جملوں کو اونچی آواز میں پڑھیں"۔ درجہ بندی حسب ذیل ہے: ۴= درست، (ث) مختصر شکل کو مکمل میں تبدیل کر دیں؛ ۳= درست، لیکن تین سیکنڈ سے زیادہ تاخیر سے، خود درست کیا ہو یا بار بار دہرایا ہو؛ ۲= (الف سے ت) کم از کم نِصف صوتیات درست ہوں یا ہدف پر اپریکزک غلطی، (ٹ اور ث) میں کم از کم نِصف الفاظ ہوں؛ ۱= ہدف سے کچھ تعلق، ٹ اور ث کے لیے دو سے زیادہ الفاظ ہوں؛ ۰= غیر متعلقہ جواب یا چھ سیکنڈ کے اندر کوئی جواب نہیں۔

الف) تنگ ______________________________ [۰][۱][۲][۳][۴]

ب) ڈھول ______________________________ [۰][۱][۲][۳][۴]

پ) پرورش ______________________________ [۰][۱][۲][۳][۴]

ت) ان پڑھ ______________________________ [۰][۱][۲][۳][۴]

ٹ) بچہ رات کو روتا ہے۔ ______________________________ [۰][۱][۲][۳][۴]

ث) مشہور ناول نگار کو احساس ہوا کہ مجھے کیوں بلایا جا رہا تھا۔ ______________________ [۰][۱][۲][۳][۴]

۸) گُفتاری حرکت

مریض سے (الف)، (پ) اور (ت) جلد از جلد انجام دینے کو کہیں۔ (ب) میں دیے گئے حرفِ عِلت کو جتنا ممکن ہو طویل کھینچیں، زیادہ سے زیادہ ۱۵ سیکنڈ تک۔ گنتی کو عام رفتار سے کیا جا سکتا ہے۔ اگر گُفتاری حرکت کی کمزوریاں مجتبی ہوں تو اضافی مشق دیں؛ مثلاً (ہونٹوں کو سکڑانا اور کھینچنا، "کیٹسٹراف کیٹسٹراف"، "/ک/ / ک/ / ک/ / ک/"، "/ٹ/ /ٹ/ /ٹ/ /ٹ/"، "کیٹسٹراف" کہنا، یا /س/ کو مسلسل لمبا کرنا۔

الف) زبان ایک طرف سے دوسری طرف لگائیں ______________________

ب) آآآآہ (آواز کا معیار) ______________________

پ) /پ/ /پ/ /پ/ /پ/ /پ/ /پ/ (شرح اور تال) ______________________

ت) /پ/ /ٹ/ /ک/ /پ/ /ٹ/ /ک/ /پ/ /ٹ/ /ک/ (شرح اور تال) ____________

ٹ) دس تک گنتی کریں۔ ______________________

گُفتاری حرکت کی درج ذیل پیمانوں کا استعمال کرتے ہوئے، نہ کہ صرف اس حصے کی بنیاد پر بلکہ پوری تشخیص کی بنیاد پر درجہ بندی کریں۔

گُفتاری حرکت کی خصوصیات	۰	۱	۲	۳	۴
ڈس آرتھریا: بولنے کے لیے استعمال ہونے والے پٹھوں کی خرابی	شدید	نمایاں	درمیانہ	معمولی	بلکل صحیح
اپریکسیا: بولنے کی حرکاتی منصوبہ بندی کی خرابی	شدید	نمایاں	درمیانہ	معمولی	بلکل صحیح

خلاصہ:

لفظ کی سمجھ ______________________________

جملے کی سمجھ ______________________________

لفظ تلاش کرنے کی کارکردگی ____________________

قواعدی جملہ سازی کی کارکردگی ____________________

گُفتاری حرکت منصوبہ بندی ____________________

دہرائی ______________________________

بلند آواز سے پڑھنا __________________________

مجموعی کیو اے بی __________________________

کوئنگ افیزیا بیٹری

فارم ۲

شریک کنندہ ____________ معالج ____________ وقت ____________

تاریخ ____________ مقام ____________

- ہر جُز کا جواب دینے کے لیے چھ سیکنڈ کا وقت دیں۔ تین سیکنڈ کے بعد جوابات کو تاخیر میں شمار کریں اور اس کے تین نمبر دیں۔ صرف پہلے چھ سیکنڈ میں دیے گئے جواب کو نمبر دیں، سوائے اس کے کہ جہاں جواب چھ سیکنڈ سے پہلے شروع کیا جائے اور پھر جاری رکھا جائے۔
- پہلے مکمل جواب کو نمبر دیں۔(یعنی ایسا جواب نہیں جس کا غلط آغاز ہو یا وہ نامکمل ہو)
- اگر مریض درخواست کرے یا معالج کو مریض کے غلط سُننے یا توجہ دینے میں کوتاہی کا شُبہ ہو تو زبانی طور پر پیش کردہ جُز کو ایک بار دہرایا جا سکتا ہے۔ اگر کوئی جُز دہرایا جاتا ہے تو چھ سیکنڈ کی گنتی کو دوبارہ شروع کریں۔
- ایسی ڈس آرتھریا یا اپریکسیا کی غلطی کو نظر انداز کریں جو کسی بھی صوتیہ کی شناخت کو متاثر نہ کر رہی ہو، سوائے اس کے کہ جب گُفتاری حرکت کی درجہ بندی کی جا رہی ہو۔

۱) شعور کی سطح

الف) کیا مریض طبی طور پر اس قدر مستحکم ہے کہ اس سے رابطہ کیا جا سکتا ہے؟ [۰(رکو)] [۴]

رابطہ کیا جا سکتا ہے؟ درجہ بندی درج ذیل ہے: ۴= ہاں؛ ۰= نہیں، نرسوں اور ڈاکٹروں وغیرہ سے فیصلے کی بنیاد پر۔

ب) کیا مریض کو بیدار کیا جا سکتا ہے؟ [۰(رکو)] [۱(رکو)] [۲] [۳] [۴]

بیدار کیا جا سکتا ہے؟ درجہ بندی درج ذیل ہے: ۴= پہلے سے بیدار ہے یا ہلکے محرک سے بیدار ہوتا ہے؛ ۳= درمیانی درجے کے محرک سے بیدار ہوتا ہے؛ ۲= بیدار ہوتا ہے، لیکن بیداری برقرار نہیں رکھتا؛ ۱= کچھ ردِ عمل دکھاتا ہے لیکن بیدار نہیں ہوتا؛ ۰= کوئی جواب نہیں۔

باشعور و باخبر درجہ بندی درج ذیل ہے: ۴= ایک قابل فہم درست جواب (کسی بھی تقریر یا زبان کی غلطیوں کو یہاں نظر انداز کیا جائے گا)؛ ۳= تینوں ہاں یا نہیں سوالات کے درست جوابات؛ ۲= ہاں سوال پر درست اور آدھے نہیں سوالات پر درست جوابات؛ ۱= سمجھنے اور جواب دینے کی کوشش کی مشابہ علامت؛ ۰= جواب اور سوال کو سمجھنے کی کوئی علامت نہیں دکھاتا ہے یا کوئی جواب نہیں دیتا۔

ت) کیا آپ مجھے بتا سکتے ہیں کہ ہم اس وقت کہاں ہیں؟ ____________________ [۰] [۱] [۲] [۳] [۴]

اگر کوئی جواب نہیں یا غلط جواب: کیا ہم لائبریری میں ہیں؟ [ہاں] [نہیں] [کوئی جواب نہیں]

کیا ہم کھیل کے میدان میں ہیں؟ [ہاں] [نہیں] [کوئی جواب نہیں]

کیا ہم [صحیح جگہ] میں ہیں؟ [ہاں] [نہیں] [کوئی جواب نہیں]

ٹ) یہ کون سا مہینہ ہے؟ ____________________ [۰] [۱] [۲] [۳] [۴]

اگر کوئی جواب نہیں یا غلط جواب: کیا یہ [غلط مہینہ] ہے؟ [ہاں] [نہیں] [کوئی جواب نہیں]

کیا یہ [غلط مہینہ] ہے؟ [ہاں] [نہیں] [کوئی جواب نہیں]

کیا یہ [درست مہینہ] ہے؟ [ہاں] [نہیں] [کوئی جواب نہیں]

ث) آپ کی عمر کتنی ہے؟ ____________________ [۰] [۱] [۲] [۳] [۴]

اگر کوئی جواب نہیں یا غلط جواب: کیا آپ [غلط عمر] کے ہیں؟ [ہاں] [نہیں] [کوئی جواب نہیں]

کیا آپ [درست عمر] کے ہیں؟ [ہاں] [نہیں] [کوئی جواب نہیں]

کیا آپ [غلط عمر] کے ہیں؟ [ہاں] [نہیں] [کوئی جواب نہیں]

احکامات کی پیروی کر سکتا ہے؟ درجہ بندی مندرجہ ذیل ہے: ۴= زبانی حکم کی پیروی کرتا ہے؛ ۳= عمل کی نقل کرتا ہے؛ ۱= زبانی حکم پر عمل کرنے یا عمل کی نقل کرنے کی مشابہ کوشش کرتا ہے؛ ۰= جواب، حکم کو سمجھنے کا کوئی نشان نہیں دکھاتا یا کوئی جواب نہیں دیتا۔ اگر حالات یا مریض کے عوامل حکم دیتے ہیں تو متبادل آسان حکم کو تبدیل کیا جا سکتا ہے، اس صورت میں انہیں نوٹ کریں۔

ج) آنکھیں بند کریں۔ ____________________ [۰][۱][۳][۴]

اگر بند نہ کریں تو: اپنی آنکھیں بند کریں اور نقل کرنے کا اشارہ کریں۔

چ) میرا ہاتھ دبائیں۔ ____________________ [۰][۱][۳][۴]

اگر نہ کریں تو: مریض کا ہاتھ دبائیں اور نقل کرنے کا اشارہ کریں۔

مجموعی تاثر: درجہ بندی درج ذیل ہے: ۴= ہاں؛ ۳= ہاں، مگر کچھ کمی پیشی (مثلاً توجہ میں وقفہ)؛ ۲= کبھی کبھی ؛ ۱ = معمولی؛ ۰= بالکل نہیں۔

ح) کیا مریض بیدار رہ سکتا ہے، توجہ برقرار رکھ سکتا ہے اور احکامات کی پیروی کر سکتا ہے؟

[۰(رکو)] [۱(رکو)] [۲(رکو)] [۳] [۴]

۲) گفتگو میں تسلسل

کم از کم تین منٹ تک شریک کندہ کے ساتھ بات چیت کریں، ان میں سے ایک یا زیادہ گفتگو کے عنوانات پر جیسے:

- جب آپ کا پہلا بچہ ہوا؟
- جب آپ نے زندگی کی سب سے پہلی سواری خریدی؟
- آپ جہاں رہتے ہیں وہاں کے بارے میں آپ کو کیا پسند ہے؟
- ایک وقت جب آپ واقع ہی خوفزدہ یا شرمندہ یا ناراض تھے؟
- بہترین سفر جو آپ نے کیا ہے؟
- سب سے بُرا سفر جو آپ نے کیا ہے؟
- جب آپ کی شادی ہوئی؟
- بچپن میں آپ کی پسندیدہ چھٹی؟
- بچپن کی کوئی خوشگوار یاد؟
- آپ کی پہلی نوکری؟
- آپ کی بچپن کی سب سے بُری یاد؟

تحریکی صفحہ ۱ پیش کریں۔ پوچھیں "یہاں کیا ہو رہا ہے؟"

الف) لڑکی لڑکے کو مار رہی ہے۔ ________________________________

ب) لڑکا لڑکی کو چُپ کروا رہا ہے۔________________________________

درج ذیل پیمانوں کا استعمال کرتے ہوئے تسلسل والی گُفتار کی درجہ بندی کریں۔ تشخیص میں تیار کی گئی دیگر تسلسل والی گُفتار پر بھی غور کریں۔ ۴= معمول کے مطابق: کوئی غلطی موجود نہیں یا عام حد کے اندر؛ ۳= معمولی: قابل شناخت لیکن کبھی کبھار؛ ۲= درمیانہ: بار بار لیکن وسیع نہیں؛ ۱= نمایاں: وسیع لیکن ہر جگہ نہیں؛ ۰= شدید: زیادہ تر یا تمام الفاظ میں واضح۔

افیزک تسلسل والی گفتگو کی خصوصیات	۰	۱	۲	۳	۴
الفاظ کی لمبائی اور پیچیدگی میں کمی	واحد لفظ	گفتگو کی اوسط لمبائی: دو الفاظ	گفتگو کی اوسط لمبائی: تین سے چار الفاظ	گفتگو کی اوسط لمبائی: پانچ سے سات الفاظ	معمول کے مطابق
بول چال کی شرح میں کمی	صفر سے چوبیس لفظ فی منٹ	پچیس سے انچاس الفاظ فی منٹ	پچاس سے چوھتر الفاظ فی منٹ	پچھتر سے ننانوے الفاظ فی منٹ	سو سے زیادہ الفاظ
بے قاعدہ گرامر: الفاظ اور صرفیات کو چھوڑنا، خاص طور پر قواعدی الفاظ؛ "مختصر جملہ بندی"	شدید	نمایاں	درمیانہ	معمولی	معمول کے مطابق

غیر مربوط گرامر: قواعدی الفاظ کا متبادل؛ الفاظ اور جملوں کا نامناسب جوڑ؛ خراب نحو	شدید	نمایاں	درمیانہ	معمولی	معمول کے مطابق
لفظ یاد آنے میں مشکل: لفظ تلاش کرنے میں وقفہ، الفاظ کی تلاش میں ناکامی، ترک شدہ الفاظ، کامیاب اشارہ کنائی	شدید	نمایاں	درمیانہ	معمولی	معمول کے مطابق
غیر مرکزی گفتگو: مبہم الفاظ، قواعدی الفاظ کی کثرت، ناکام اشارہ کنائی، لفظوں کا غیر واضح معنی	شدید	نمایاں	درمیانہ	معمولی	معمول کے مطابق
معنوی لفظی تبدیلیاں: کھلے زمرے کے الفاظ کا متبادل، بار بار پچھلے لفظ کی دہرائی	شدید	نمایاں	درمیانہ	معمولی	معمول کے مطابق
صوتی لفظی تبدیلیاں اور خود ساختہ الفاظ	شدید	نمایاں	درمیانہ	معمولی	معمول کے مطابق
خود درستگی: غلط آغاز، روکی گئی یا دوبارہ شروع کی گئی جملہ بندی، خود اصلاحی کوشش	شدید	نمایاں	درمیانہ	معمولی	معمول کے مطابق

مجموعی طور پر گفتگو میں خرابی	گفتگو ٹکڑوں میں ہو: گفتگو قائم رکھنے کی مکمل ذمہ داری ممتحن پر ہو۔	محدود گفتگو ہو: شریک کندھا بھی گفتگو قائم کرنے کی ذمہ داری لے رہا ہو۔	روزمرہ کے موضوعات پر گفتگو کر سکتا ہو؛ مگر اس سے باہر گفتگو محدود ہو۔	خرابی واضح ہو، مگر گفتگو تمام موضوعات پر کر سکتا ہو۔	کوئی خرابی واضح نہ ہو۔

اگر ممکن نہ ہو:

- کوئی خود ساختہ گفتگو نہیں
- صرف بے ربط بڑبڑاہٹ
- صرف بار بار دہرائے جانے والے مخصوص جملے یا الفاظ
- دس الفاظ فی منٹ سے کم، بنیادی طور پر صرف ہاں یا نہ، چند ایک الفاظ یا ہلکی سی کوشش

۳) لفظ کی سمجھ

تحریکی صفحہ ۲ پیش کریں۔ کہیں "مجھے دکھائیں"۔ درجہ بندی حسب ذیل ہے: ۴= درست؛ ۳= درست لیکن تین سیکنڈ کی تاخیر سے زیادہ، خود درستگی یا بار بار دہرائی؛ ۱= متعلقہ ورق؛ ۰= متعلقہ متبادل ورق یا چھ سیکنڈ کے اندر کوئی جواب نہیں۔

الف) قمیض متعلقہ: شلوار، دستانے ________________ [۴][۳][۱][۰]

ب) چیتا متعلقہ: زیبرا، گدھا ________________ [۴][۳][۱][۰]

پ) زیبرا متعلقہ: چیتا، گدھا ________________ [۴][۳][۱][۰]

ت) شلوار متعلقہ: دستانے، قمیض ________________ [۴][۳][۱][۰]

تحریکی صفحہ ۳ پیش کریں۔

ٹ) کان متعلقہ: پان ________________ [۴][۳][۱][۰]

ث) نان متعلقہ: دکان ________________________________ [۰][۱][۳][۴]

ج) پان متعلقہ: قرآن ________________________________ [۰][۱][۳][۴]

چ) زبان متعلقہ: کان ________________________________ [۰][۱][۳][۴]

۴) جملے کی سمجھ

کہیں "ہاں یا نہیں" میں جواب دیں۔ درجہ بندی حسب ذیل ہے: ۴ = درست سرہلانا، ہاں یا نہیں کے دیگر سیاق و سباق کے مطابق مناسب متبادل جیسے "کبھی کبھی"، "مجھے ایسا نہیں لگتا"، یا غیر معمولی ردِ عمل جو سمجھ کا مظاہرہ کرتے ہیں قابل قبول ہیں؛ ۳ = درست، لیکن، تین سیکنڈ سے زیادہ تاخیر، خوددرستگی یا بار بار دہرائی؛ ۲ = غیر معمولی ردِ عمل جہاں یہ واضح نہیں ہے کہ مریض سمجھتا ہے یا نہیں۔ ۱ = غلط، لیکن جواب کچھ جزوی فہم کی تجویز کرتا ہے؛ ۰ = غلط یا چھ سیکنڈ کے اندر کوئی جواب نہیں۔

الف) کیا آپ [بیٹھے/لیٹے/ وغیرہ] ہیں؟ (ہاں) ____________________________ [۰][۱][۲][۳][۴]

ب) کیا میں [بیٹھا/کھڑا/ وغیرہ] ہوں؟ (ہاں) ____________________________ [۰][۱][۲][۳][۴]

پ) کیا آپ آئس کریم چمچ سے کھاتے ہیں؟ (ہاں) ____________________________ [۰][۱][۲][۳][۴]

ت) کیا لکڑیوں کو لوگ کاٹتے ہیں؟ (نہیں) ____________________________ [۰][۱][۲][۳][۴]

ٹ) کیا آپ اپنے پیروں پر دستانے پہنتے ہیں؟ (نہیں) ____________________________ [۰][۱][۲][۳][۴]

ث) اگر آپ باہر جانے والے ہیں کیا آپ ابھی بھی اندر ہیں؟ (ہاں) ________________ [۰][۱][۲][۳][۴]

ج) کیا پرندے کیڑے کھاتے ہیں؟ (ہاں) ____________________________ [۰][۱][۲][۳][۴]

چ) اگر میں آپ کو بتاؤں کہ میں ورزش کرتا تھا، تو کیا آپ کو لگتا ہے کہ میں اب بھی ورزش کرتا ہوں؟

(نہیں) ____________________________ [۰][۱][۲][۳][۴]

ح) کیا ڈاکٹر بچوں کا علاج کرتے ہیں؟ (ہاں) ____________________________ [۰][۱][۲][۳][۴]

خ) اگر آپ شروع کرنے والے ہیں، کیا آپ نے ابھی تک شروع کیا ہے؟ (نہیں) __________ [۰][۱][۲][۳][۴]

ڈ) کیا بچے والدین کی پرورش کرتے ہیں؟ (نہیں) ____________ [۰][۱][۲][۳][۴]

ڈ) اگر آپ پارک میں تھے جب میں پہنچا تھا، تو کیا آپ وہاں پہلے پہنچے تھے؟ (ہاں) ____________ [۰][۱][۲][۳][۴]

۵) تصویر کو نام دینا

تحریکی صفحہ ۵ پیش کریں اور پوچھیں "یہ کیا ہے؟"۔ درج بندی حسب ذیل ہے: ۴= قابل قبول درست جواب؛ ۳= درست، لیکن تین سیکنڈ سے زیادہ تاخیر سے یا خود درست کیا ہو؛ ۲= کم از کم نِصف صوتیات درست ہوں یا ہدف پر اپریکزک غلطی؛ ۱= ہدف سے کچھ تعلق؛ ۰= غیر متعلقہ جواب یا چھ سیکنڈ کے اندر کوئی جواب نہیں۔

الف) کتاب ____________ [۰][۱][۲][۳][۴]

ب) کنگھی ____________ [۰][۱][۲][۳][۴]

پ) ٹوپی ____________ [۰][۱][۲][۳][۴]

ت) آتش فشاں ____________ [۰][۱][۲][۳][۴]

ٹ) گھوڑا ____________ [۰][۱][۲][۳][۴]

ث) پہاڑ ____________ [۰][۱][۲][۳][۴]

٦) دہرائی:

کہیں "میرے بعد دہرائیں"۔ درجہ بندی حسب ذیل ہے: ۴= درست، (ث) مختصر شکل کو مکمل میں تبدیل کر دیں؛ ۳= درست، لیکن تین سیکنڈ سے زیادہ تاخیر سے، خود درست کیا ہو یا بار بار دہرایا ہو؛ ۲= (الف سے ت) کم از کم نِصف صوتیات درست ہوں یا ہدف پر اپریکزک غلطی، (ٹ اور ث) میں کم از کم نِصف الفاظ ہوں؛ ۱= ہدف سے کچھ تعلق، ٹ اور ث کے لیے دو سے زیادہ الفاظ ہوں؛ ۰= غیر متعلقہ جواب یا چھ سیکنڈ کے اندر کوئی جواب نہیں۔

الف) آم ____________ [۰][۱][۲][۳][۴]

ب) بلکل ____________ [۰][۱][۲][۳][۴]

پ) پرورش ____________ [۰][۱][۲][۳][۴]

ت) انگریزی ______________________ [۰][۱][۲][۳][۴]

ٹ) کتا دروازے پر بھونکتا ہے۔ ______________________ [۰][۱][۲][۳][۴]

ث) تخلیقی معمار سمجھ گیا کہ ہم کس کو دیکھیں گے۔ ______________________ [۰][۱][۲][۳][۴]

۷) بلند آواز سے پڑھنا

تحریکی صفحہ ٦ پیش کریں۔ کہیں "ان الفاظ اور جملوں کو اونچی آواز میں پڑھیں"۔ درجہ بندی حسب ذیل ہے:۴= درست،(ث) مختصر شکل کو مکمل میں تبدیل کر دیں؛ ۳= درست، لیکن تین سیکنڈ سے زیادہ تاخیر سے، خود درست کیا ہو یا بار بار دہرایا ہو؛ ۲= (الف سے ت) کم از کم نِصف صوتیات درست ہوں یا ہدف پر اپریکز ک غلطی،(ٹ اور ث)میں کم از کم نِصف الفاظ ہوں؛ ۱=ہدف سے کچھ تعلق، ٹ اور ث کے لیے دو سے زیادہ الفاظ ہوں؛ ۰= غیر متعلقہ جواب یا چھ سیکنڈ کے اندر کوئی جواب نہیں۔

الف) بندر ______________________ [۰][۱][۲][۳][۴]

ب) کلونجی ______________________ [۰][۱][۲][۳][۴]

پ) گردہ ______________________ [۰][۱][۲][۳][۴]

ت) غیر معمولی ______________________ [۰][۱][۲][۳][۴]

ٹ) سورج مغرب میں غروب ہوتا ہے۔ ______________________ [۰][۱][۲][۳][۴]

ث) قابل جاسوس نے دریافت کیا کہ میں کیوں انتظار کر رہا تھا۔ ______________________ [۰][۱][۲][۳][۴]

۸) گُفتاری حرکت

مریض سے (الف)، (پ) اور (ت) جلد از جلد انجام دینے کو کہیں۔ (ب) میں دیے گئے حرف عِلت کو جتنا ممکن ہو طویل کھینچیں، زیادہ سے زیادہ ۱۵ سیکنڈ تک۔ گنتی کو عام رفتار سے کیا جا سکتا ہے۔ اگر گُفتاری حرکت کی کمزوریاں مجتبی ہوں تو اضافی مشق دیں؛ مثلاً (ہونٹوں کو سکڑا نا اور کھینچنا، "کیٹسٹراف کیٹسٹراف"، "/ک/ / ک/ / ک/ /ک/"، "/ٹ/ /ٹ/ / ٹ/ /ٹ/"، "کیٹسٹراف" کہنا، یا /س/ کو مسلسل لمبا کرنا.

الف) زبان ایک طرف سے دوسری طرف لگائیں ____________________

ب) آآآآہ (آواز کا معیار) ____________________

پ) /پ/ /پ/ /پ/ /پ/ /پ/ /پ/ (شرح اور تال) ____________________

ت) /پ/ /ٹ/ /ک/ /پ/ /ٹ/ /ک/ /پ/ /ٹ/ /ک/ (شرح اور تال) __________

ٹ) دس تک گنتی کریں۔ ____________________

گُفتاری حرکت کی درج ذیل پیمانوں کا استعمال کرتے ہوئے، نہ کہ صرف اس حصے کی بنیاد پر بلکہ پوری تشخیص کی بنیاد پر درجہ بندی کریں۔

گُفتاری حرکت کی خصوصیات	۰	۱	۲	۳	۴
ڈس آرتھریا: بولنے کے لیے استعمال ہونے والے پٹھوں کی خرابی	شدید	نمایاں	درمیانہ	معمولی	بلکل صحیح
اپریکسیا: بولنے کی حرکاتی منصوبہ بندی کی خرابی	شدید	نمایاں	درمیانہ	معمولی	بلکل صحیح

خلاصہ:

لفظ کی سمجھ ________________________

جملے کی سمجھ ________________________

لفظ تلاش کرنے کی کارکردگی ________________

قواعدی جملہ سازی کی کارکردگی ________________

گُفتاری حرکت منصوبہ بندی ________________

دہرائی ________________________

بلند آواز سے پڑھنا ________________________

مجموعی کیو اے بی ________________________

کومَک افیزیا بیٹری

فارم ۳

شریک کنندہ ____________ معالج ____________ وقت ____________

تاریخ ____________ مقام ____________

- ہر جُز کا جواب دینے کے لیے چھ سیکنڈ کا وقت دیں۔ تین سیکنڈ کے بعد جوابات کو تاخیر میں شمار کریں اور اس کے تین نمبر دیں۔ صرف پہلے چھ سیکنڈ میں دیے گئے جواب کو نمبر دیں، سوائے اس کے کہ جہاں جواب چھ سیکنڈ سے پہلے شروع کیا جائے اور پھر جاری رکھا جائے۔
- پہلے مکمل جواب کو نمبر دیں۔(یعنی ایسا جواب نہیں جس کا غلط آغاز ہو یا وہ نامکمل ہو)
- اگر مریض درخواست کرے یا معالج کو مریض کے غلط سُننے یا توجہ دینے میں کوتاہی کا شُبہ ہو تو زبانی طور پر پیش کردہ جُز کو ایک بار دہرایا جا سکتا ہے۔ اگر کوئی جُز دہرایا جاتا ہے تو چھ سیکنڈ کی گنتی کو دوبارہ شروع کریں۔
- ایسی ڈِس آرتھریا یا اپریکسیا کی غلطی کو نظر انداز کریں جو کسی بھی صوتیہ کی شناخت کو متاثر نہ کر رہی ہو، سوائے اس کے کہ جب گُفتاری حرکت کی درجہ بندی کی جا رہی ہو۔

۱) شعور کی سطح

الف) کیا مریض طبی طور پر اس قدر مستحکم ہے کہ اس سے رابطہ کیا جا سکتا ہے؟ [۰ (رکو)] [۴]

رابطہ کیا جا سکتا ہے؟ درجہ بندی درج ذیل ہے: ۴= ہاں؛ ۰ = نہیں، نرسوں اور ڈاکٹروں وغیرہ سے فیصلے کی بنیاد پر۔

ب) کیا مریض کو بیدار کیا جا سکتا ہے؟ [۰(رکو)] [۱(رکو)] [۲] [۳] [۴]

بیدار کیا جا سکتا ہے؟ درجہ بندی درج ذیل ہے: ۴= پہلے سے بیدار ہے یا ہلکے محرک سے بیدار ہوتا ہے؛ ۳= درمیانی درجے کے محرک سے بیدار ہوتا ہے؛ ۲= بیدار ہوتا ہے، لیکن بیداری برقرار نہیں رکھتا؛ ۱= کچھ ردِ عمل دکھاتا ہے لیکن بیدار نہیں ہوتا؛ ۰= کوئی جواب نہیں۔

باشعور و باخبر درجہ بندی درج ذیل ہے: ۴= ایک قابل فہم درست جواب (کسی بھی تقریر یا زبان کی غلطیوں کو یہاں نظر انداز کیا جائے گا)؛ ۳= تینوں ہاں یا نہیں سوالات کے درست جوابات؛ ۲= ہاں سوال پر درست اور آدھے نہیں سوالات پر درست جوابات؛ ۱ = سمجھنے اور جواب دینے کی کوشش کی مشابہ علامت؛ ۰= جواب اور سوال کو سمجھنے کی کوئی علامت نہیں دکھاتا ہے یا کوئی جواب نہیں دیتا۔

ت) کیا آپ مجھے بتا سکتے ہیں کہ ہم اس وقت کہاں ہیں؟ ____________________ [۰] [۱] [۲] [۳] [۴]

اگر کوئی جواب نہیں یا غلط جواب: کیا ہم لائبریری میں ہیں؟ [ہاں] [نہیں] [کوئی جواب نہیں]

کیا ہم کھیل کے میدان میں ہیں؟ [ہاں] [نہیں] [کوئی جواب نہیں]

کیا ہم [صحیح جگہ] میں ہیں؟ [ہاں] [نہیں] [کوئی جواب نہیں]

ٹ) یہ کون سا مہینہ ہے؟ ____________________ [۰] [۱] [۲] [۳] [۴]

اگر کوئی جواب نہیں یا غلط جواب: کیا یہ [غلط مہینہ] ہے؟ [ہاں] [نہیں] [کوئی جواب نہیں]

کیا یہ [غلط مہینہ] ہے؟ [ہاں] [نہیں] [کوئی جواب نہیں]

کیا یہ [درست مہینہ] ہے؟ [ہاں] [نہیں] [کوئی جواب نہیں]

ث) آپ کی عمر کتنی ہے؟ ____________________ [۰] [۱] [۲] [۳] [۴]

اگر کوئی جواب نہیں یا غلط جواب: کیا آپ [غلط عمر] کے ہیں؟ [ہاں] [نہیں] [کوئی جواب نہیں]

کیا آپ [درست عمر] کے ہیں؟ [ہاں] [نہیں] [کوئی جواب نہیں]

کیا آپ [غلط عمر] کے ہیں؟ [ہاں] [نہیں] [کوئی جواب نہیں]

احکامات کی پیروی کر سکتا ہے؟ درجہ بندی مندرجہ ذیل ہے: ۴= زبانی حکم کی پیروی کرتا ہے؛ ۳= عمل کی نقل کرتا ہے؛ ۱= زبانی حکم پر عمل کرنے یا عمل کی نقل کرنے کی مشابہ کوشش کرتا ہے؛ ۰= جواب، حکم کو سمجھنے کا کوئی نشان نہیں دکھاتا یا کوئی جواب نہیں دیتا۔ اگر حالات یا مریض کے عوامل حکم دیتے ہیں تو متبادل آسان حکم کو تبدیل کیا جا سکتا ہے، اس صورت میں انہیں نوٹ کریں۔

ج) آنکھیں بند کریں۔ ____________________ [۰][۱][۳][۴]

اگر بند نہ کریں تو: اپنی آنکھیں بند کریں اور نقل کرنے کا اشارہ کریں۔

چ) میرا ہاتھ دبائیں۔ ______________________ [۰][۱][۳][۴]

اگر نہ کریں تو: مریض کا ہاتھ دبائیں اور نقل کرنے کا اشارہ کریں۔

مجموعی تاثر: درجہ بندی درج ذیل ہے: ۴= ہاں؛ ۳= ہاں، مگر کچھ کمی پیشی (مثلاً توجہ میں وقفہ)؛ ۲= کبھی کبھی ؛ ۱= معمولی؛ ۰= بالکل نہیں۔

ح) کیا مریض بیدار رہ سکتا ہے، توجہ برقرار رکھ سکتا ہے اور احکامات کی پیروی کر سکتا ہے؟

[۰(رکو)] [۱(رکو)] [۲(رکو)] [۳] [۴]

۲) گفتگو میں تسلسل

کم از کم تین منٹ تک شریک کنندہ کے ساتھ بات چیت کریں، ان میں سے ایک یا زیادہ گفتگو کے عنوانات پر جیسے:

- جب آپ کا پہلا بچہ ہوا؟
- جب آپ نے زندگی کی سب سے پہلی سواری خریدی؟
- آپ جہاں رہتے ہیں وہاں کے بارے میں آپ کو کیا پسند ہے؟
- ایک وقت جب آپ واقع ہی خوفزدہ یا شرمندہ یا ناراض تھے؟
- بہترین سفر جو آپ نے کیا ہے؟
- سب سے بُرا سفر جو آپ نے کیا ہے؟
- جب آپ کی شادی ہوئی؟
- بچپن میں آپ کی پسندیدہ چھٹی؟
- بچپن کی کوئی خوشگوار یاد؟
- آپ کی پہلی نوکری؟
- آپ کی بچپن کی سب سے بُری یاد؟

تحریکی صفحہ ۱پیش کریں۔ پوچھیں "یہاں کیا ہو رہا ہے؟"

الف) لڑکی لڑکے کو کھینچ رہی ہے۔ ________________________________

ب) لڑکا لڑکی کو جگا رہا ہے۔________________________________

درج ذیل پیمانوں کا استعمال کرتے ہوئے تسلسل والی گُفتار کی درجہ بندی کریں۔ تشخیص میں تیار کی گئی دیگر تسلسل والی گُفتار پر بھی غور کریں۔ ۴= معمول کے مطابق: کوئی غلطی موجود نہیں یا عام حد کے اندر؛ ۳= معمولی: قابل شناخت لیکن کبھی کبھار؛ ۲= درمیانہ: بار بار لیکن وسیع نہیں؛ ۱= نمایاں: وسیع لیکن ہر جگہ نہیں؛ ۰= شدید: زیادہ تر یا تمام الفاظ میں واضح۔

افیزک تسلسل والی گفتگو کی خصوصیات	۰	۱	۲	۳	۴
الفاظ کی لمبائی اور پیچیدگی میں کمی	واحد لفظ	گفتگو کی اوسط لمبائی: دو الفاظ	گفتگو کی اوسط لمبائی: تین سے چار الفاظ	گفتگو کی اوسط لمبائی: پانچ سے سات الفاظ	معمول کے مطابق
بول چال کی شرح میں کمی	صفر سے چوبیس لفظ فی منٹ	پچیس سے انچاس الفاظ فی منٹ	پچاس سے چوہتر الفاظ فی منٹ	پچھتر سے ننانوے الفاظ فی منٹ	سو سے زیادہ الفاظ
بے قاعدہ گرامر: الفاظ اور صرفیات کو چھوڑنا، خاص طور پر قواعدی الفاظ؛ "مختصر جملہ بندی"	شدید	نمایاں	درمیانہ	معمولی	معمول کے مطابق

غیر مربوط گرامر: قواعدی الفاظ کا متبادل؛ الفاظ اور جملوں کا نامناسب جوڑ؛ خراب نحو	شدید	نمایاں	درمیانہ	معمولی	معمول کے مطابق
لفظ یاد آنے میں مشکل: لفظ تلاش کرنے میں وقفہ، الفاظ کی تلاش میں ناکامی، ترک شدہ الفاظ، کامیاب اشارہ کنائی	شدید	نمایاں	درمیانہ	معمولی	معمول کے مطابق
غیر مرکزی گفتگو: مبہم الفاظ، قواعدی الفاظ کی کثرت، ناکام اشارہ کنائی، لفظوں کا غیر واضح معنی	شدید	نمایاں	درمیانہ	معمولی	معمول کے مطابق
معنوی لفظی تبدیلیاں: کھلے زمرے کے الفاظ کا متبادل، بار بار پچھلے لفظ کی دہرائی	شدید	نمایاں	درمیانہ	معمولی	معمول کے مطابق
صوتی لفظی تبدیلیاں اور خود ساختہ الفاظ	شدید	نمایاں	درمیانہ	معمولی	معمول کے مطابق
خود درستگی: غلط آغاز، روکی گئی یا دوبارہ شروع کی گئی جملہ بندی، خود اصلاحی کوشش	شدید	نمایاں	درمیانہ	معمولی	معمول کے مطابق

مجموعی طور پر گفتگو میں خرابی	گفتگو ٹکڑوں میں ہو: گفتگو قائم رکھنے کی مکمل ذمہ داری ممتحن پر ہو۔	محدود گفتگو ہو: شریک کندھا بھی گفتگو قائم کرنے کی ذمہ داری لے رہا ہو۔	روزمرہ کے موضوعات پر گفتگو کر سکتا ہو؛ مگر اس سے باہر گفتگو محدود ہو۔	خرابی واضح ہو، مگر گفتگو تمام موضوعات پر کر سکتا ہو۔	کوئی خرابی واضح نہ ہو۔

اگر ممکن نہ ہو:

- کوئی خود ساختہ گفتگو نہیں
- صرف بے ربط بڑبڑاہٹ
- صرف بار بار دہرائے جانے والے مخصوص جملے یا الفاظ
- دس الفاظ فی منٹ سے کم، بنیادی طور پر صرف ہاں یا نہ، چند ایک الفاظ یا ہلکی سی کوشش

۳) لفظ کی سمجھ

تحریکی صفحہ ۲ پیش کریں۔ کہیں "مجھے دکھائیں"۔ درج بندی حسب ذیل ہے: ۴= درست؛ ۳= درست لیکن تین سیکنڈ کی تاخیر سے زیادہ، خوددرستگی یا بار بار دہرائی؛ ۱= متعلقہ ورق؛ ۰= متعلقہ متبادل ورق یا چھ سیکنڈ کے اندر کوئی جواب نہیں۔

الف) ہاتھی متعلقہ: اونٹ، ریچھ ________________ [۴][۳][۱][۰]

ب) کلہاڑا متعلقہ: ہتھوڑی، رسی ________________ [۴][۳][۱][۰]

پ) اونٹ متعلقہ: ہاتھی، ریچھ ________________ [۴][۳][۱][۰]

ت) ہتھوڑی متعلقہ: کلہاڑا، رسی ________________ [۴][۳][۱][۰]

تحریکی صفحہ ۳ پیش کریں۔

ٹ) اونٹ متعلقہ: ہونٹ ______________________ [۰][۱][۳][۴]

ث) چوٹ متعلقہ: نوٹ ______________________ [۰][۱][۳][۴]

ج) کوٹ متعلقہ: اخروٹ ______________________ [۰][۱][۳][۴]

چ) نوٹ متعلقہ: ہونٹ ______________________ [۰][۱][۳][۴]

۴) جملے کی سمجھ

کہیں "ہاں یا نہیں" میں جواب دیں۔ درجہ بندی حسب ذیل ہے: ۴ = درست سرہلانا، ہاں یا نہیں کے دیگر سیاق و سباق کے مطابق مناسب متبادل جیسے "کبھی کبھی"، "مجھے ایسا نہیں لگتا"، یا غیر معمولی رِد عمل جو سمجھ کا مظاہرہ کرتے ہیں قابلِ قبول ہیں؛ ۳= درست، لیکن، تین سیکنڈ سے زیادہ تاخیر، خود درستگی یا بار بار دہرائی؛ ۲= غیر معمولی رِد عمل جہاں یہ واضح نہیں ہے کہ مریض سمجھتا ہے یا نہیں۔ ۱ = غلط، لیکن جواب کچھ جزوی فہم کی تجویز کرتا ہے؛ ۰= غلط یا چھ سیکنڈ کے اندر کوئی جواب نہیں۔

الف) کیا میں نے ایک [رنگ][قمیض یا لباس]پہن رکھا یا رکھی ہے؟ (ہاں) ______________ [۰][۱][۲][۳][۴]

ب) کیا میں نے ایک [رنگ][قمیض یا لباس]پہن رکھا یا رکھی ہے؟ (نہیں) ______________ [۰][۱][۲][۳][۴]

پ) کیا آپ اپنے دانتوں کو کنگھی سے صاف کرتے ہیں؟ (نہیں) ______________ [۰][۱][۲][۳][۴]

ت) کیا بچوں کے نام والدین رکھتے ہیں؟ (ہاں) ______________ [۰][۱][۲][۳][۴]

ٹ) کیا آپ کیمرے سے تصویر لیتے ہیں؟ (ہاں) ______________ [۰][۱][۲][۳][۴]

ث) اگر آپ ختم کرنے والے ہیں، کیا آپ نے ابھی تک ختم کر لیا ہے؟ (نہیں) ______________ [۰][۱][۲][۳][۴]

ج) کیا حکومتوں کے ذریعے لوگوں پر ٹیکس لگایا جاتا ہے؟ (ہاں) ______________ [۰][۱][۲][۳][۴]

چ) اگر آپ اندر جانے والے ہیں تو کیا اپ ابھی بھی باہر ہیں؟ (ہاں) ______________ [۰][۱][۲][۳][۴]

ح) کیا بھیڑیوں پر ہرن حملے کرتے ہیں؟ (نہیں) ________________ [۰][۱][۲][۳][۴]

خ) اگر وہ شو میں تھا جب آپ پہنچے تو کیا وہ پہلے وہاں تھا؟ (ہاں) ________________ [۰][۱][۲][۳][۴]

د) کیا متاثرین چوروں کو لوٹتے ہیں؟ (نہیں) ________________ [۰][۱][۲][۳][۴]

ڈ) اگر میں آپ کو بتاؤں کہ میں کافی پیتا تھا تو کیا آپ کو لگتا ہے کہ میں اب بھی کافی پیتا ہوں؟

(نہیں) ________________ [۰][۱][۲][۳][۴]

۵) تصویر کو نام دینا

تحریکی صفحہ ۵ پیش کریں اور پوچھیں "یہ کیا ہے؟"۔ درجہ بندی حسب ذیل ہے: ۴= قابلِ قبول درست جواب؛ ۳= درست، لیکن تین سیکنڈ سے زیادہ تاخیر سے یا خود درست کیا ہو؛ ۲= کم از کم نِصف صوتیات درست ہوں یا ہدف پر اپریکزک غلطی؛ ۱= ہدف سے کچھ تعلق؛ ۰= غیر متعلقہ جواب یا چھ سیکنڈ کے اندر کوئی جواب نہیں۔

الف) پلنگ ________________ [۰][۱][۲][۳][۴]

ب) پھول ________________ [۰][۱][۲][۳][۴]

پ) درخت ________________ [۰][۱][۲][۳][۴]

ت) چھری ________________ [۰][۱][۲][۳][۴]

ٹ) کبوتر ________________ [۰][۱][۲][۳][۴]

ث) ٹیکہ ________________ [۰][۱][۲][۳][۴]

٦) دہرائی:

کہیں میرے بعد دہرائیں۔ درج بندی حسب ذیل ہے: ۴= درست، (ث) مختصر شکل کو مکمل میں تبدیل کر دیں؛ ۳= درست، لیکن تین سیکنڈ سے زیادہ تاخیر سے، خود درست کیا ہویا بار بار دہرایا ہو؛ ۲= (الف سے ت) کم از کم نِصف صوتیات درست ہوں یا ہدف پر اپریکز ک غلطی، (ٹ اور ث) میں کم از کم نِصف الفاظ ہوں؛ ۱= ہدف سے کچھ تعلق، ٹ اور ث کے لیے دو سے زیادہ الفاظ ہوں؛ ۰= غیر متعلقہ جواب یا چھ سیکنڈ کے اندر کوئی جواب نہیں۔

الف) ہڈی ______________________ [۰][۱][۲][۳][۴]

ب) برادری ______________________ [۰][۱][۲][۳][۴]

پ) پروانہ ______________________ [۰][۱][۲][۳][۴]

ت) انگیٹھی ______________________ [۰][۱][۲][۳][۴]

ٹ) بچہ بوتل سے دودھ پیتا ہے۔ ______________________ [۰][۱][۲][۳][۴]

ث) پر اعتماد گلوکار کو احساس ہوا کہ ہم کہاں رہ رہے ہوں گے۔ ______________ [۰][۱][۲][۳][۴]

۷) بلند آواز سے پڑھنا

تحریکی صفحہ ٦ پیش کریں۔ کہیں "ان الفاظ اور جملوں کو اونچی آواز میں پڑھیں"۔ درجہ بندی حسب ذیل ہے: ۴= درست، (ث) مختصر شکل کو مکمل میں تبدیل کر دیں؛ ۳= درست، لیکن تین سیکنڈ سے زیادہ تاخیر سے، خود درست کیا ہویا بار بار دہرایا ہو؛ ۲= (الف سے ت) کم از کم نِصف صوتیات درست ہوں یا ہدف پر اپریکز ک غلطی، (ٹ اور ث) میں کم از کم نِصف الفاظ ہوں؛ ۱= ہدف سے کچھ تعلق، ٹ اور ث کے لیے دو سے زیادہ الفاظ ہوں؛ ۰= غیر متعلقہ جواب یا چھ سیکنڈ کے اندر کوئی جواب نہیں۔

الف) ٹانگ ______________________ [۰][۱][۲][۳][۴]

ب) کھانسی ______________________ [۰][۱][۲][۳][۴]

پ) پرورش ______________________ [۰][۱][۲][۳][۴]

ت) ان پڑھ ______________________ [۰][۱][۲][۳][۴]

ٹ) کتا فرش پر سوتا ہے۔ ______________________ [۴][۳][۲][۱][۰]

ث) اخلاقی محاسب سمجھ گیا کہ میں کیوں چھپا رہا ہوں گا۔ ______________________ [۴][۳][۲][۱][۰]

۸) گُفتاری حرکت

مریض سے (الف)، (پ) اور (ت) جلد از جلد انجام دینے کو کہیں۔ (ب) میں دیے گئے حرف عِلت کو جتنا ممکن ہو طویل کھینچیں، زیادہ سے زیادہ ۱۵ سیکنڈ تک۔ گنتی کو عام رفتار سے کیا جا سکتا ہے۔ اگر گُفتاری حرکت کی کمزوریاں مجتبی ہوں تو اضافی مشق دیں؛ مثلاً (ہونٹوں کو سکڑانا اور کھینچنا، "کیٹسٹراف کیٹسٹراف"، "/ک/ /ک/ /ک/ /ٹ/"، "/ٹ/ /ٹ/"، "کیٹسٹراف" کہنا، یا /س/ کو مسلسل لمبا کرنا.

الف) زبان ایک طرف سے دوسری طرف لگائیں ______________________

ب) آآآآہ (آواز کا معیار) ______________________

پ) /پ/ /پ/ /پ/ /پ/ /پ/ (شرح اور تال) ______________________

ت) /پ/ /ٹ/ /ک/ /پ/ /ٹ/ /ک/ /پ/ /ٹ/ /ک/ (شرح اور تال) ______________________

ٹ) دس تک گنتی کریں۔ ______________________

گُفتاری حرکت کی درج ذیل پیمانوں کا استعمال کرتے ہوئے، نہ کہ صرف اس حصے کی بنیاد پر بلکہ پوری تشخیص کی بنیاد پر درجہ بندی کریں۔

گُفتاری حرکت کی خصوصیات	۰	۱	۲	۳	۴
ڈس آرتھریا: بولنے کے لیے استعمال ہونے والے پٹھوں کی خرابی	شدید	نمایاں	درمیانہ	معمولی	بلکل صحیح
اپریکسیا: بولنے کی حرکاتی منصوبہ بندی کی خرابی	شدید	نمایاں	درمیانہ	معمولی	بلکل صحیح

خلاصہ:

لفظ کی سمجھ ______________________

جملے کی سمجھ ______________________

لفظ تلاش کرنے کی کارکردگی ______________________

قواعدی جملہ سازی کی کارکردگی ______________________

گُفتاری حرکت منصوبہ بندی ______________________

دہرائی ______________________

بلند آواز سے پڑھنا ______________________

مجموعی کیو اے بی ______________________
