# Supplementary material for: Adaptation of the Quick Aphasia Battery into Urdu and establishment of its psychometric properties
Source: PLoS One. 2026 Sep 1;21(9):e0356908. doi: 10.1371/journal.pone.0356908 (PMC13533341; doi:10.1371/journal.pone.0356908)
Supplement: Supplementary file 1 [file pone.0356908.s001.zip › QAB-Urdu/QAB stimulus card/┌⌐┘ê┘è┘ö┌⌐ ╪o ┘ü█î╪▓█î╪o ╪¿█î┘╣╪▒█î 02.pdf]

کوئک افیزیا بیٹری

فارم ۲

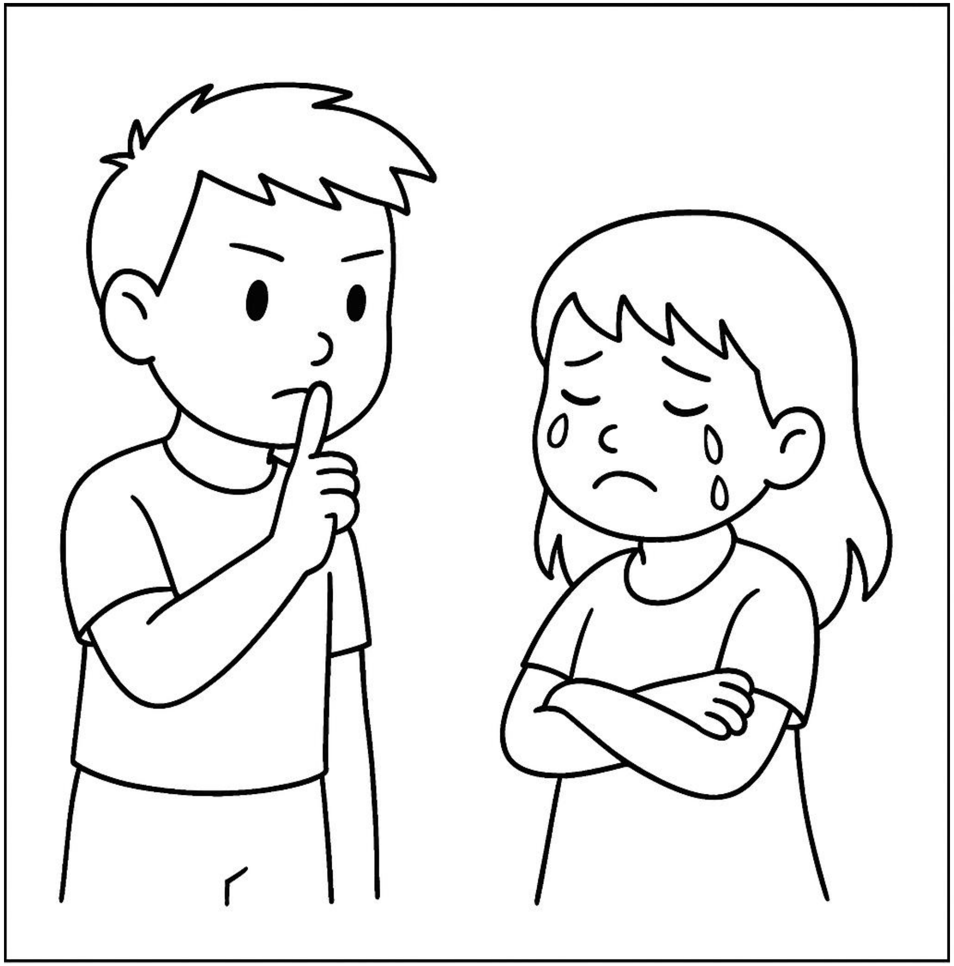

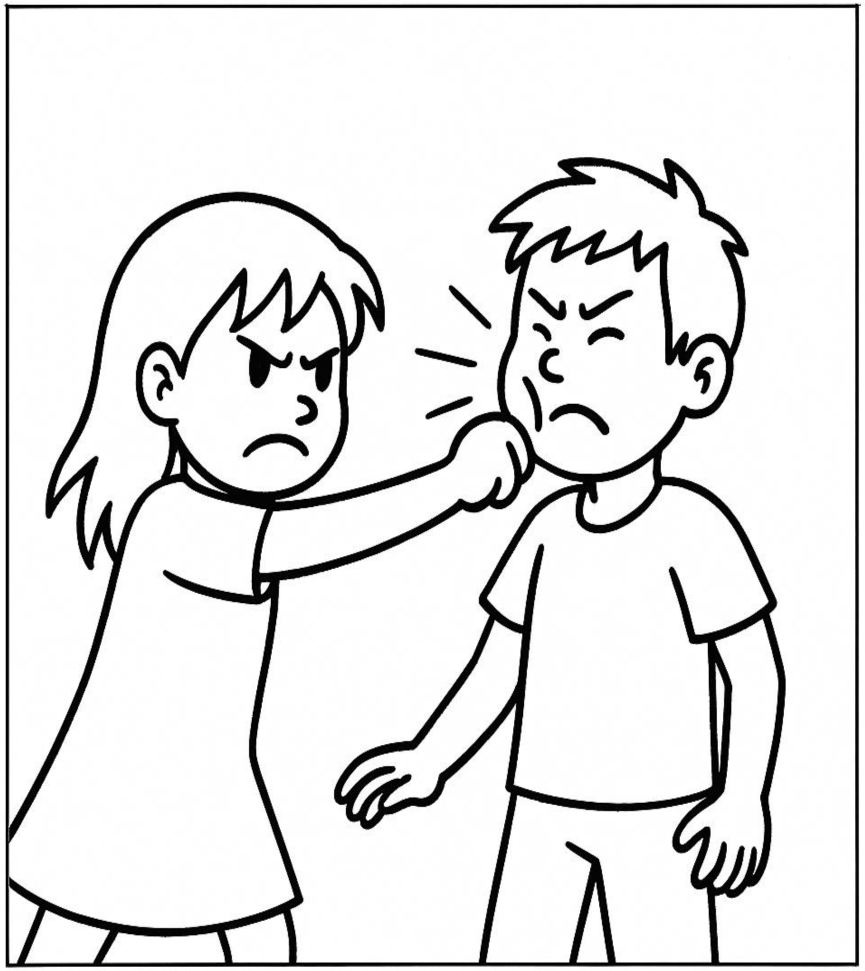

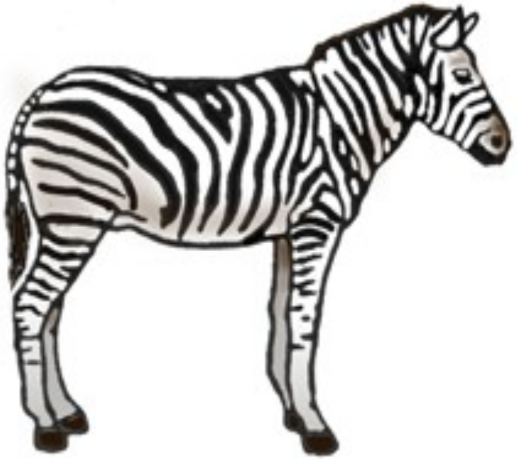

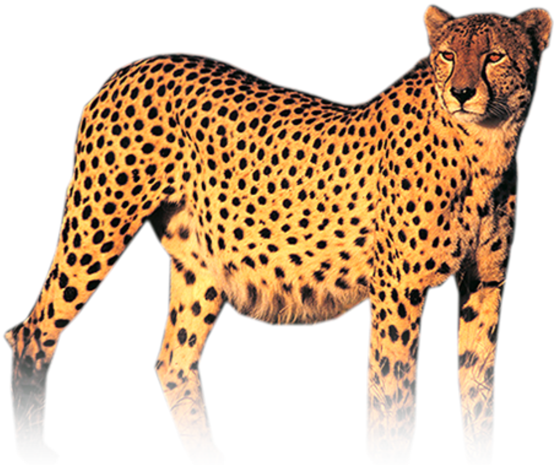

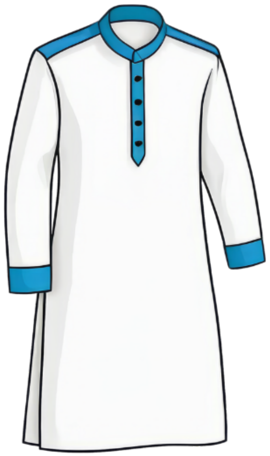

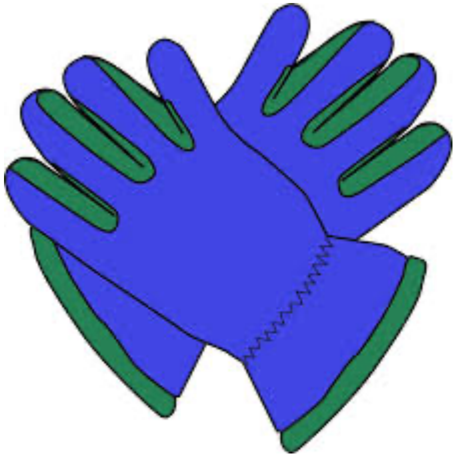

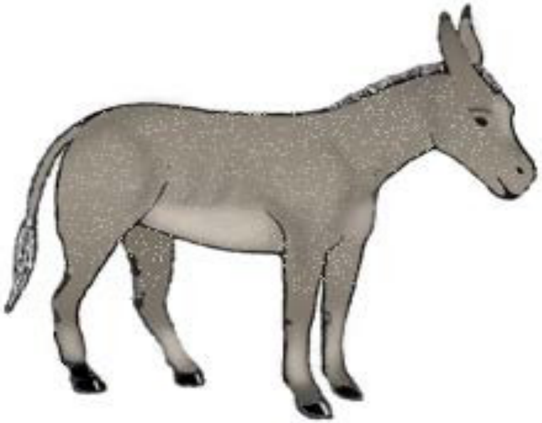

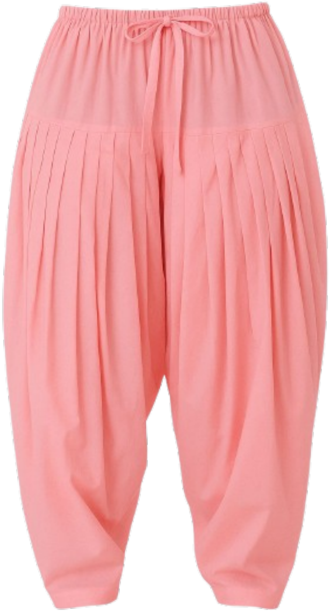

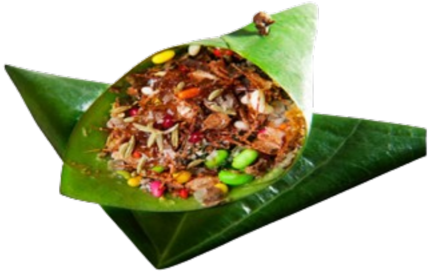
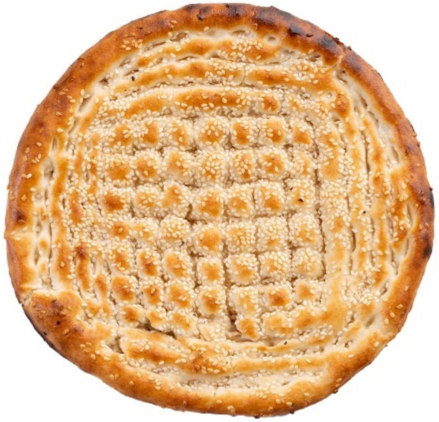
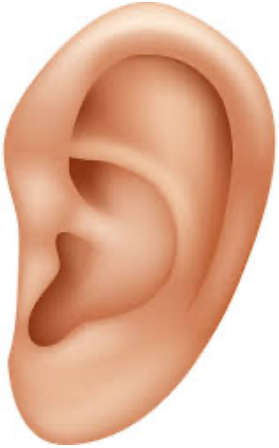
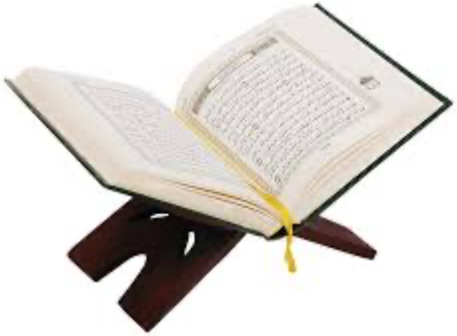
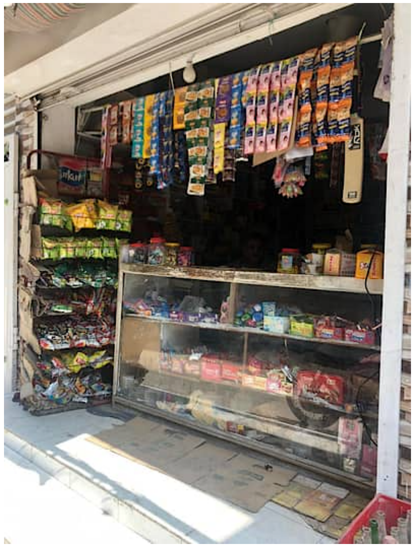
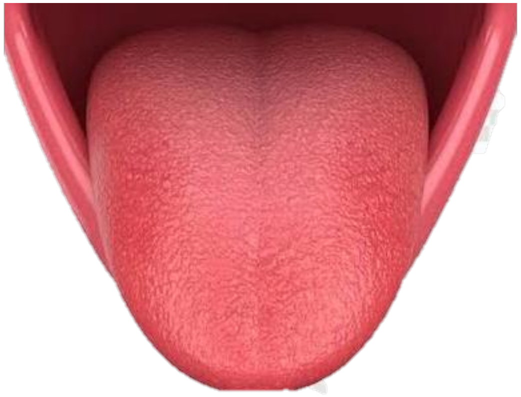

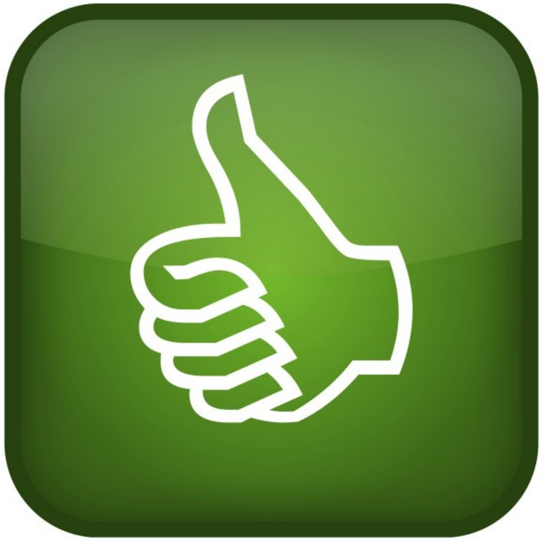

ہاں

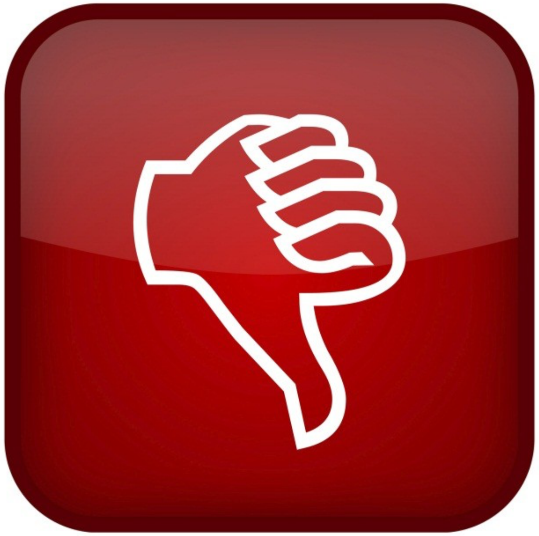

نہیں

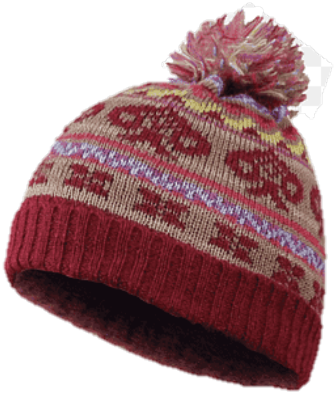
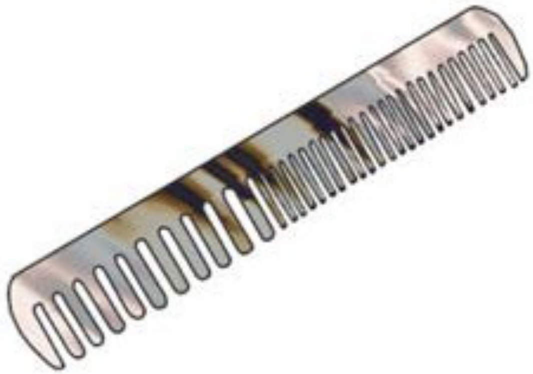
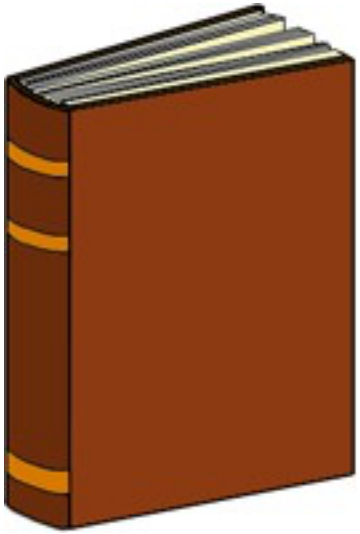
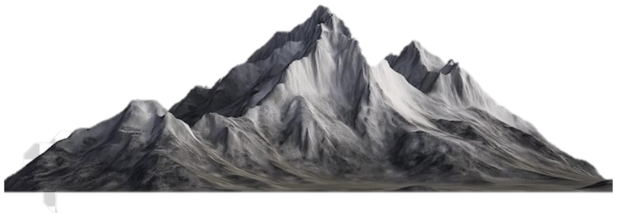
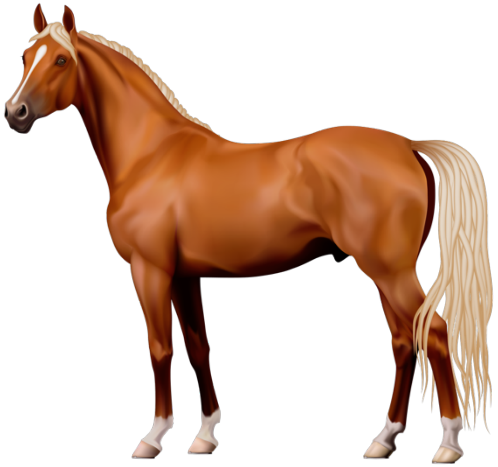
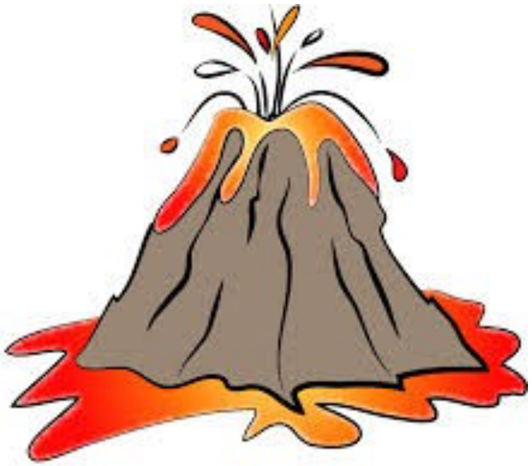

بندر

کلونجی

گردہ

غیر معمولی

سورج مغرب میں غروب ہوتا ہے.

قابل جاسوس نے دریافت کیا کہ میں کیوں انتظار کر رہا تھا.
