# Supplementary material for: Adaptation of the Quick Aphasia Battery into Urdu and establishment of its psychometric properties
Source: PLoS One. 2026 Sep 1;21(9):e0356908. doi: 10.1371/journal.pone.0356908 (PMC13533341; doi:10.1371/journal.pone.0356908)
Supplement: Supplementary file 1 [file pone.0356908.s001.zip › QAB-Urdu/QAB stimulus card/┌⌐┘ê┘è┘ö┌⌐ ╪o ┘ü█î╪▓█î╪o ╪¿█î┘╣╪▒█î03.pdf]

کوئک افیزیا بیٹری

فارم ۳

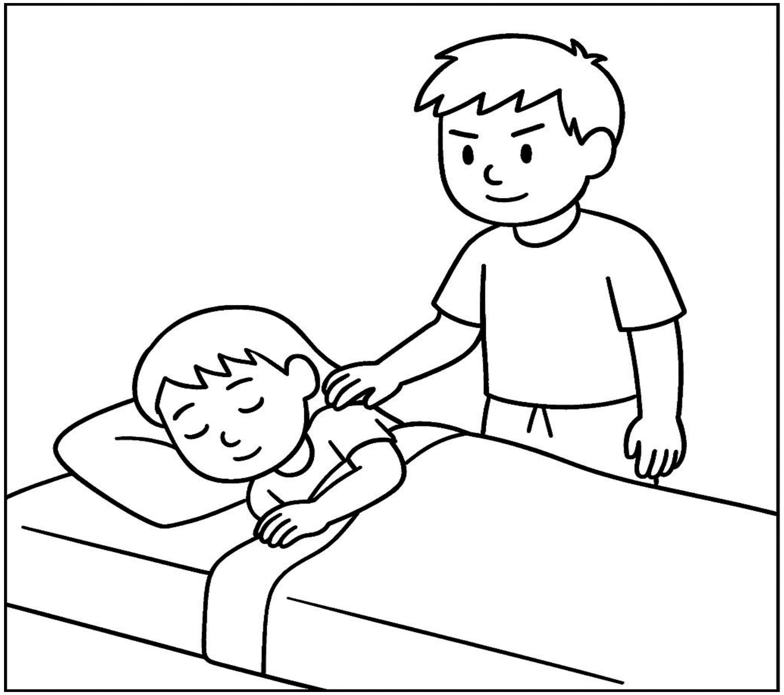

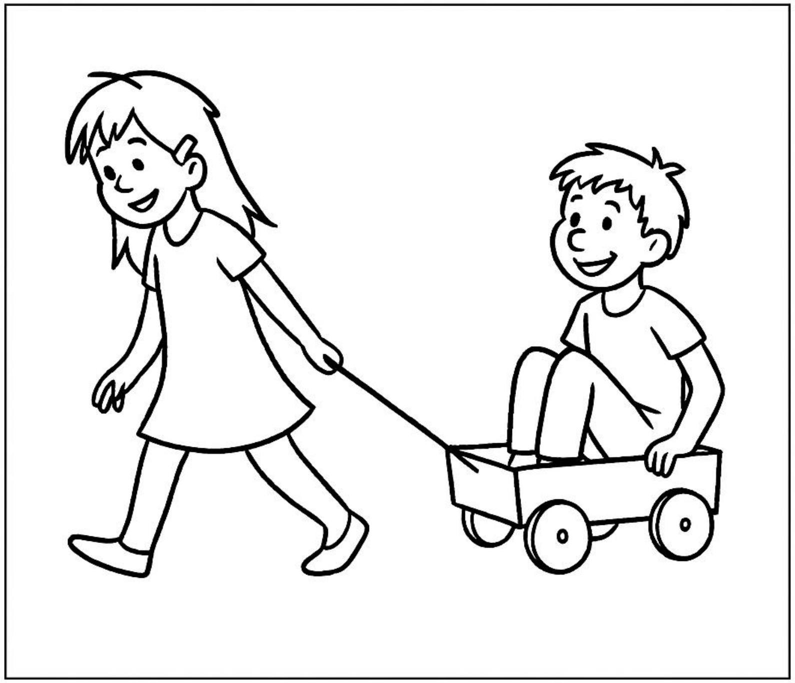

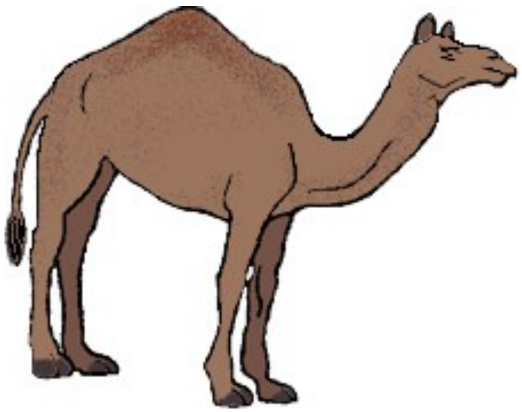
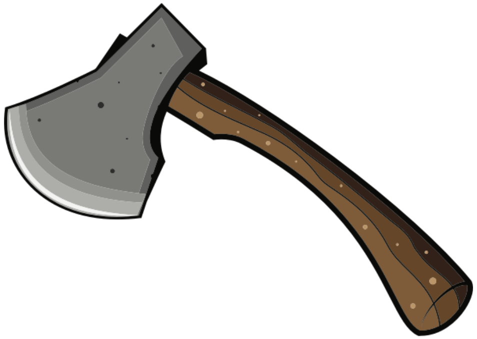
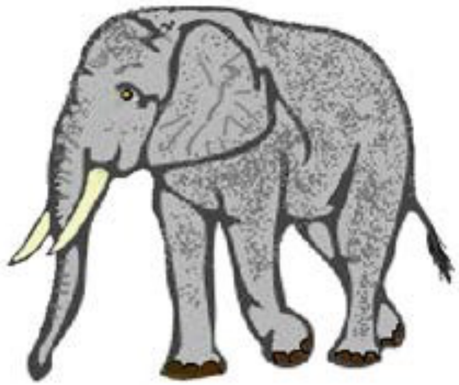
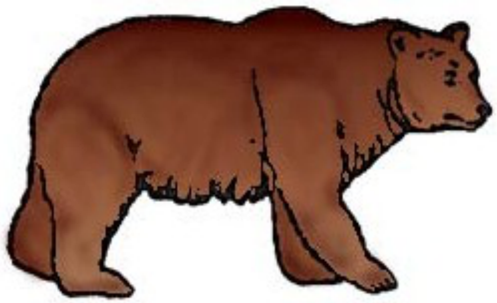
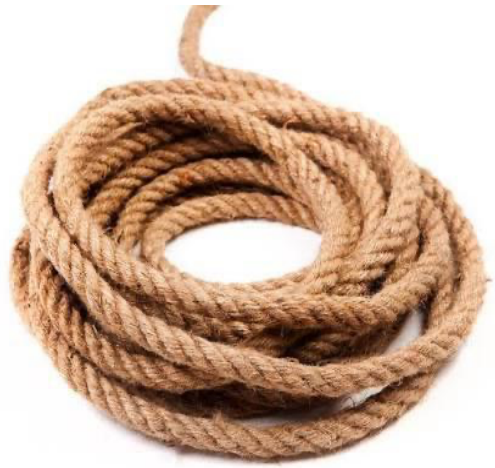
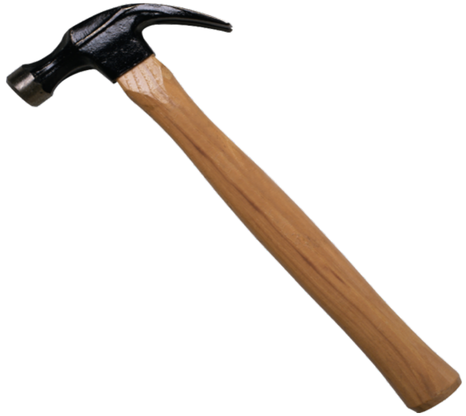

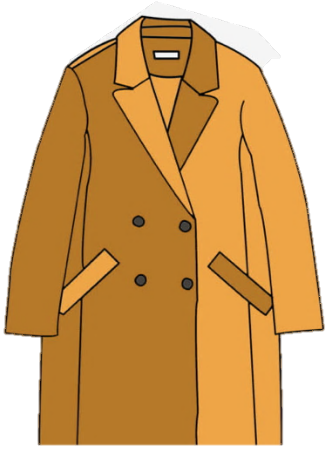

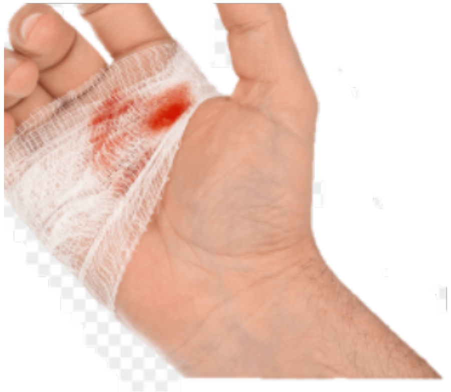

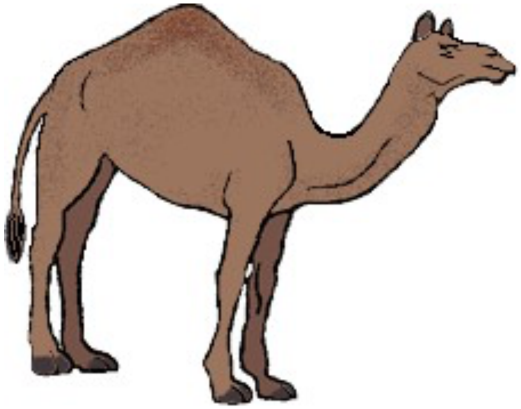

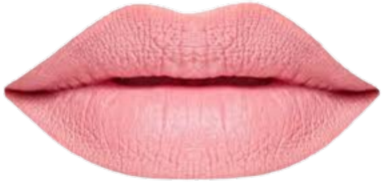

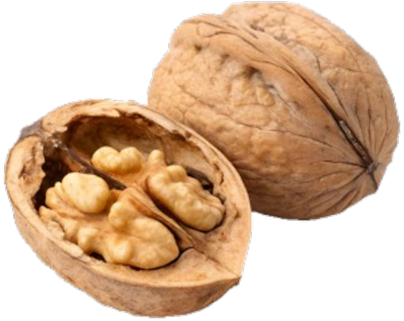

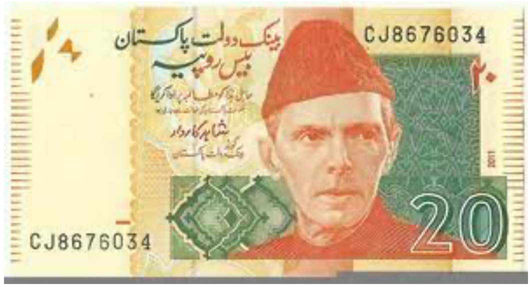
بینک دولت پاکستان
بیس روپیہ
CJ8676034
CJ8676034
20

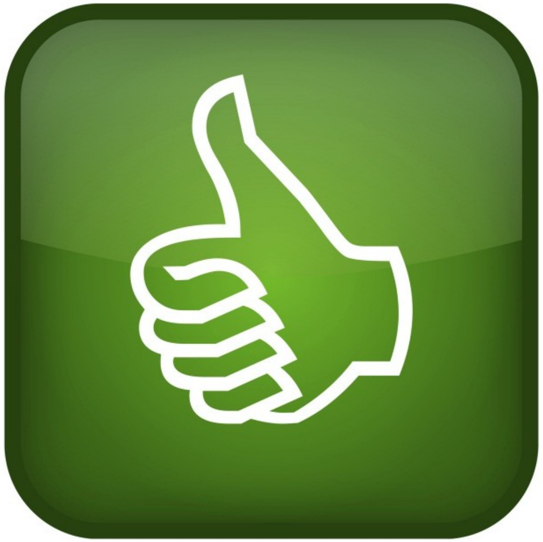

ہاں

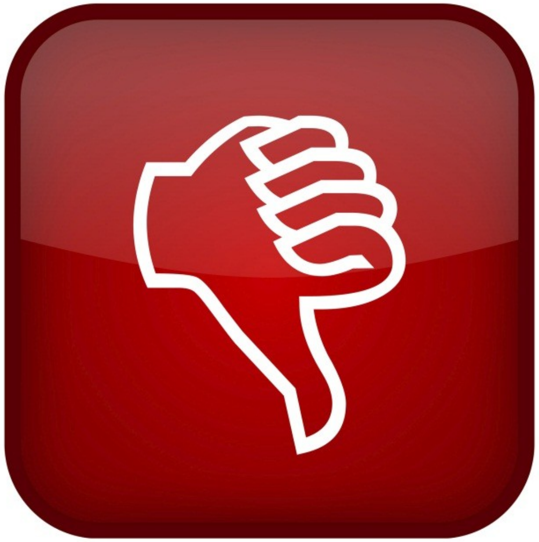

نہیں

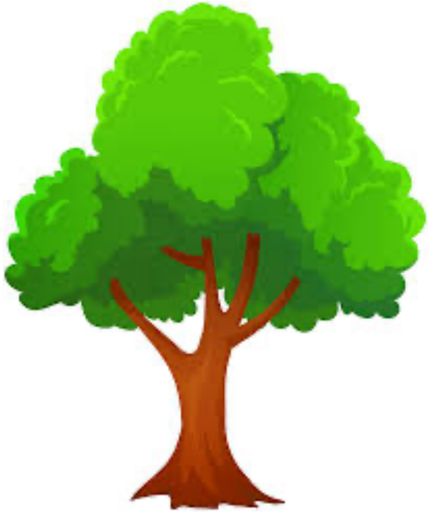

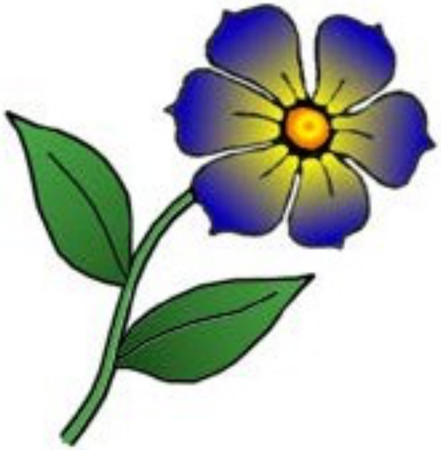

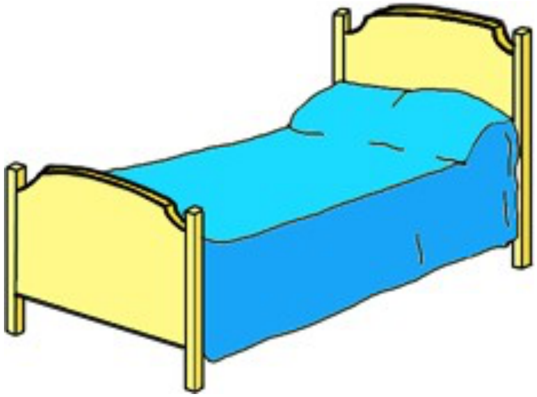

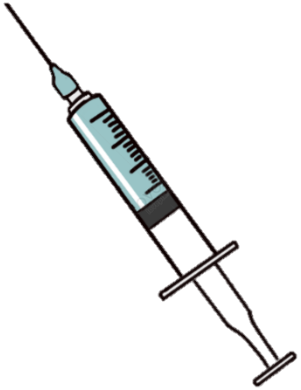

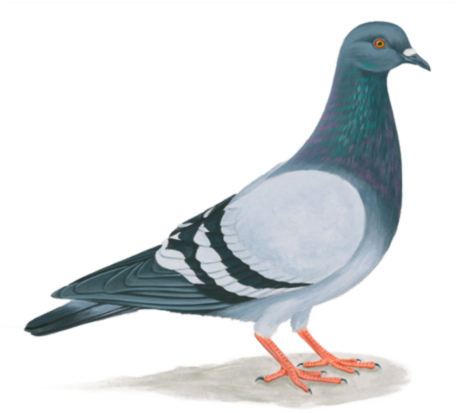

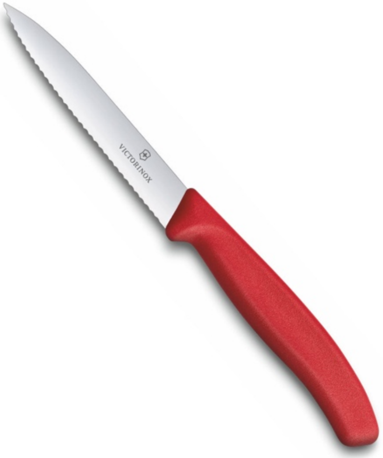
VICTORINOX

ٹانگ

کھانسی

پرورش

ان پڑھ

کُتا فرش پر سوتا ہے۔

اخلاقی محاسب سمجھ گیا کہ میں کیوں چھپا رہا ہوں گا۔
